# Paradoxical effects of DNA tumor virus oncogenes on epithelium-derived tumor cell fate during tumor progression and chemotherapy response

**DOI:** 10.1038/s41392-021-00787-x

**Published:** 2021-11-26

**Authors:** Jiang He, Liyu Liu, Feiyu Tang, You Zhou, Huan Liu, Can Lu, Deyun Feng, Hong Zhu, Yitao Mao, Zhi Li, Lu Zhang, Yuemei Duan, Zhi Xiao, Musheng Zeng, Liang Weng, Lun-Quan Sun

**Affiliations:** 1 Department of Oncology, Xiangya Cancer Center, Xiangya Hospital, Central South University, Changsha, 410008 China; 2 Key Laboratory of Molecular Radiation Oncology Hunan Province, Changsha, 410008 China; 3 Department of Pathology, Tongji Medical College Union Hospital, Huazhong University of Science and Technology, Wuhan, 430022 China; 4 Department of Pathology, Xiangya Hospital, Central South University, Changsha, 410078 China; 5 Department of Radiology, Xiangya Hospital, Central South University, Changsha, 410078 China; 6 Institute of Gerontological Cancer Research, National Clinical Research Center for Gerontology, Changsha, 410008 China; 7 Department of Breast Surgery, Xiangya Hospital, Central South University, Changsha, 410078 China; 8 State Key Laboratory of Oncology in South China, Collaborative Innovation Center for Cancer Medicine, Sun Yat-Sen University Cancer Center, Guangzhou, 510060 China; 9 Hunan International Science and Technology Collaboration Base of Precision Medicine for Cancer, Changsha, 410008 China; 10 Center for Molecular Imaging of Central South University, Xiangya Hospital, Changsha, 410008 China

**Keywords:** Tumour virus infections, Cancer

## Abstract

Epstein-Barr virus (EBV) and human papillomavirus (HPV) infection is the risk factors for nasopharyngeal carcinoma and cervical carcinoma, respectively. However, clinical analyses demonstrate that EBV or HPV is associated with improved response of patients, although underlying mechanism remains unclear. Here, we reported that the oncoproteins of DNA viruses, such as LMP1 of EBV and E7 of HPV, inhibit PERK activity in cancer cells via the interaction of the viral oncoproteins with PERK through a conserved motif. Inhibition of PERK led to increased level of reactive oxygen species (ROS) that promoted tumor and enhanced the efficacy of chemotherapy in vivo. Consistently, disruption of viral oncoprotein-PERK interactions attenuated tumor growth and chemotherapy in both cancer cells and tumor-bearing mouse models. Our findings uncovered a paradoxical effect of DNA tumor virus oncoproteins on tumors and highlighted that targeting PERK might be an attractive strategy for the treatment of NPC and cervical carcinoma.

## Introduction

Approximately 12% of human cancers worldwide are caused by oncovirus infection. Despite the prevalence of oncoviruses, understanding and managing virus-induced cancers still face formidable challenges.^1,2^ DNA tumor viruses, such as Epstein-Barr virus (EBV) and human papillomavirus (HPV), are an important class of oncoviruses that can integrate into the patient genome, resulting in tumorigenesis.^3^ Mechanistically, oncoproteins, such as latent membrane protein 1 (LMP1) encoded by EBV, can transform cells via activation of nuclear factor κB (NF-κB) signaling, leading to nasopharyngeal carcinoma (NPC), Burkitt’s lymphoma, Hodgkin’s lymphomas and gastric carcinomas.^4^ The E6 and E7 oncoproteins encoded by HPV transform cells by inhibiting the functions of p53 and Rb, giving rise to the development of cervical, anal, and skin cancers.^3^ Surprisingly, although DNA tumor viruses have strong transforming abilities that can promote cancer progression, patients with DNA tumor virus-positive cancer, including HPV-positive cervical cancer and EBV-positive gastric adenocarcinoma or classical Hodgkin lymphoma (cHL), have a better prognosis than patients with virus-negative cancer.^5–8^ To date, the underlying mechanism has not been elucidated.

The unfolded protein response (UPR) is an adaptive response that can promote cell survival or trigger apoptosis.^9–11^ At least three distinct UPR signaling pathways, including the IRE1/XBP1, ATF6, and PERK-eif2a pathways, are involved in the regulation of endoplasmic reticulum (ER) stress in mammalian cells.^9^ The PERK-mediated UPR, as a key mediator in the response to stress stimuli, can facilitate or suppress malignant transformation depending on the context.^12–16^ Severe or prolonged activation of PERK attenuates cancer cell proliferation due to translation inhibition and cell cycle arrest.^12,13,16,17^ Moderate inhibition or activation of PERK contributes to cancer cells survival and proliferation.^18,19^ Therefore, PERK can function as either a tumor suppressor or a proadaptive tumor promoter depending on the gene dosage. In addition, it has been reported that the inhibition of PERK can sensitize cancer cells to chemotherapy.^20,21^ Although both the UPR and DNA tumor viruses play critical roles in tumorigenesis, whether the PERK-mediated UPR is involved in the progression and treatment response of DNA tumor virus-positive cancer is still unknown.

PERK also is involved in regulation of oxidative stress.^9^ ROS may potentially be deleterious or beneficial for cancer cells, depending on the amount of ROS production.^22–25^ A appropriate increase in ROS promotes cancer cell proliferation and tumor formation by inducing redox-dependent and pro-oncogenic signaling pathways,^26,27^ whereas excessive amounts of ROS result in oxidative damage to macromolecular, including lipids, proteins, and DNA, to trigger cancer cell death.^28,29^ Thus, a tightly controlled redox balance is critical for cancer cells function and survival. Moreover, modulation of the ROS level in cancer cells can increase sensibility to anticancer drugs.^28,30,31^ Consequently, antioxidant inhibitors prove to be a promising therapeutic strategy in anticancer therapy. Although previous studies demonstrated that EBV and HPV increased oxidative stress of cancer cells,^3,32^ the underlying mechanism and whether EBV or HPV-induced oxidative stress is involved in the progression of DNA tumor virus-positive cancer remains elusive.

In the present study, we investigated the effect of DNA tumor virus oncoproteins on the PERK-mediated UPR, tumor progression, and treatment response. We demonstrated that LMP1 of EBV and E7 of HPV interacted with PERK through a conserved transmembrane motif, which subsequently inhibited PERK oligomerization and activity. Furthermore, LMP1 and E7 enhanced cancer cell proliferation and sensitivity to anti-cancer drugs by modulating PERK activity and cellular reactive oxygen species (ROS) levels both in vitro and in vivo. Our study, for the first time, reveals that the dual effects of DNA tumor virus oncogenes on tumor progression and chemotherapy response are mediated by the PERK, which indicates that targeting PERK may serve as an attractive therapeutic strategy for NPC and cervical cancer treatment.

## Results

### DNA tumor virus oncogenes regulate PERK signaling

**Fig. 1:**
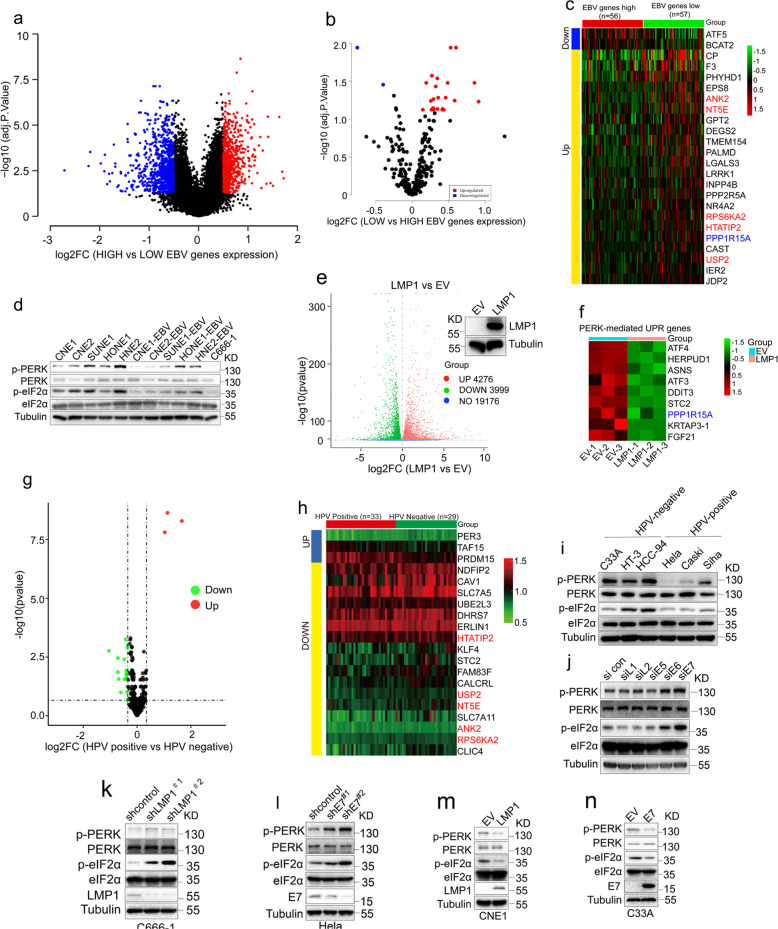
DNA tumor virus oncogenes regulate PERK activity. **a** A volcano of differentially expressed genes identified by mRNA-seq using NPC tissues with high or low EBV genes expression. **b** A volcano of differentially expressed PERK downstream genes identified by mRNA-seq using NPC tissues with high EBV genes expression or low. **c** A heat map showing the expression of the PERK downstream genes across 56 NPC samples with high EBV genes expression and 57 NPC samples with low EBV genes expression. Candidate genes based on false discovery rate cutoff of 1.2-fold change were showed. The genes highlighted with green and red color are overlapped with (**f**) and (**h**), respectively. **d** Immunoblotting to examine PERK activity in NPC cells with or without EBV infection. **e** A volcano of differentially expressed genes identified by mRNA-seq using CNE1 cells stably expressing LMP1 or an empty vector (EV). **f** Heat maps generated from RNA-seq data (from RNA-seq FPKM values) showed that LMP1 downregulated expression of PERK-mediated UPR genes. *n* = 3 independent replicates. **g** A volcano of differentially expressed PERK downstream genes identified by mRNA-seq using HPV-positive cancer samples and HPV-negative cancer samples (GSE6791). **h** A heat map showing the expression of the PERK downstream genes across HPV-positive and HPV-negative cancer samples. Candidate genes based on false discovery rate cutoff of 1-fold change were showed. **i** Immunoblotting to examine PERK activity in HPV-positive and HPV-negative cervical cancer cells. **j** HeLa cells transfected with the indicated siRNA and then evaluated by immunoblotting to monitor PERK activity. **k**, **l** C666-1 (**k**) or HeLa cells (**l**) transduced with two independent shRNAs targeting LMP1 or E7 and then evaluated by immunoblotting to determine PERK activity. **m**, **n** CNE-1 (**m**) or C33A cells (**n**) transfected with the indicated plasmids and then evaluated by immunoblotting to examine PERK activity

EBV can persistently infect and transform human cells, which induces the development of several types of cancers, such as NPC, lymphomas and gastric carcinomas. Several studies, including our studies, have shown that EBV-encoded oncoprotein LMP1 and non-coding RNA (EBER) promote NPC progression.^33,34^ To investigate the molecular events by which EBV promotes the progression of NPC, we analyzed RNA sequencing (RNA-seq) data of 113 NPC patients (GSE102349) (Fig. 1a). Based on the expression level of EBV gene, the above data were divided into EBV gene high expression and low expression groups. When the ~400 genes^10^ downstream of PERK were analyzed, up to 22 genes in low EBV gene expression group showed at least a 1.2-fold increase in expression levels including PPP1R15A, a downstream gene of PERK-mediated UPR (Fig. 1b, c and Supplementary Table S2), suggesting that the high expression of EBV genes suppresses PERK activity. To further confirm the effect of EBV on the PERK activity, we established persistent latent EBV infection in five EBV-negative malignant nasopharyngeal epithelial cell lines (CNE1, CNE2, SUNE1, HONE1, HNE2) with a recombinant vector carrying a neomycin resistance gene and then examined the levels of phosphorylated PERK and eIF2α (p-PERK and p-eIF2α), which are widely used as readouts for the activation of PERK. EBV infection led to a markedly decrease in the p-PERK and p-eIF2α levels in 4 of the 5 cell lines (Fig. 1d), among which only one nasopharyngeal epithelial cell line, HONE1-EBV, exhibited comparable levels of p-PERK and p-eIF2α to those in its EBV-negative counterpart. In addition, C666-1 cells, an EBV-positive malignant nasopharyngeal epithelial cell line, showed the decreased p-PERK and p-eIF2α levels (Fig. 1d). These results suggested that EBV infection inhibited PERK activity. Given that oncoprotein LMP1 is essential for the ability of EBV to immortalize human cells, we speculated that LMP1 may be involved in regulation of UPR. To test the hypothesis, we performed RNA-seq analysis for CNE1 cells (a NPC cell line) stably transfected with LMP1 or an empty vector (EV) (hereafter named CNE1-LMP1 and CNE1-EV cells, respectively). LMP1 expression caused significant changes in transcriptional profiles in CNE1-LMP1, in which 4699 genes upregulated and 4609 genes downregulated, including some previously reported LMP1-regulated genes (Fig. 1e and Supplementary Fig. 1a). Interestingly, when the ~400 genes^10^ downstream of PERK were further analyzed by GO enrichment analysis, the expression of PERK-mediated UPR genes was found to be inhibited in CNE1-LMP1 cells (Fig. 1f and Supplementary Fig. 1b, c). In line with previous reports,^35^ the expression of XBP1-mediated UPR genes were upregulated in CNE1-LMP1 cells (Supplementary Fig. 1d). Surprisingly, we found that LMP1 did not significantly activate IRE1-XBP1 pathway (Supplementary Fig. 1e).

To check whether other oncogenic DNA viruses also exert similar inhibitory functions on the PERK-mediated UPR pathway, we tested the roles of HPV, which is an oncogenic DNA virus associated with cervical, anal and skin cancers. We analyzed the differentially PERK downstream genes in a dataset (GSE6791) from the head/neck and cervical cancers with or without HPV infection. PERK-mediated UPR genes were downregulated in HPV positive cancers (Fig. 1g, h), some of which were overlapped with those observed in EBV positive NPC samples (Fig. 1c). These data suggested the expression of PERK downstream UPR genes is suppressed in HPV-positive cervical cancer. In cervical cancer cells, HPV-positive cervical cancer cells exhibited the decreased p-PERK and p-eIF2α levels (Fig. 1i), further supporting that that HPV infection also negatively regulates PERK activity. Although gene listed in Fig. 1c and h did not overlap completely, which may be due to the difference of HPV and EBV infection and tumor microenvironment, these data indicated that HPV and EBV can negatively regulate PERK pathway.

HPV-infected cervical cancer cells express a panel of genes, including L1, L2, E5, E6, and E7 genes. To identify which virus genes are responsible for PERK activity inhibition, we used small interfering RNA (siRNA) to knockdown the expression of HPV-encoded genes in HeLa cells (Supplementary Fig. 1f), which is positive for HPV 18 infection. We found E7 significantly decreased the levels of p-PERK and p-eIF2α (Fig. 1j). Next, we performed both loss-of-function and gain-of-function analyses targeting these two viral oncogenes. Depletion of LMP1 or E7 using two different short hairpin RNAs (shRNAs) increased the levels of p-PERK and p-eif2α in C666-1 or HeLa cells, respectively (Fig. 1k and l). Consistently, overexpression of LMP1 or E7 decreased the levels of p-PERK and p-eIF2a in CNE1 and C33A cells, which are EBV- and HPV-negative cells respectively (Fig. 1m and n). Taken together, these results show that the DNA tumor viruses EBV and HPV block the PERK-eIF2a pathway in cancer cells, which may be mediated mainly by the oncoproteins LMP1 and E7, respectively.

### DNA tumor virus oncoproteins inhibit the PERK-mediated UPR

**Fig. 2:**
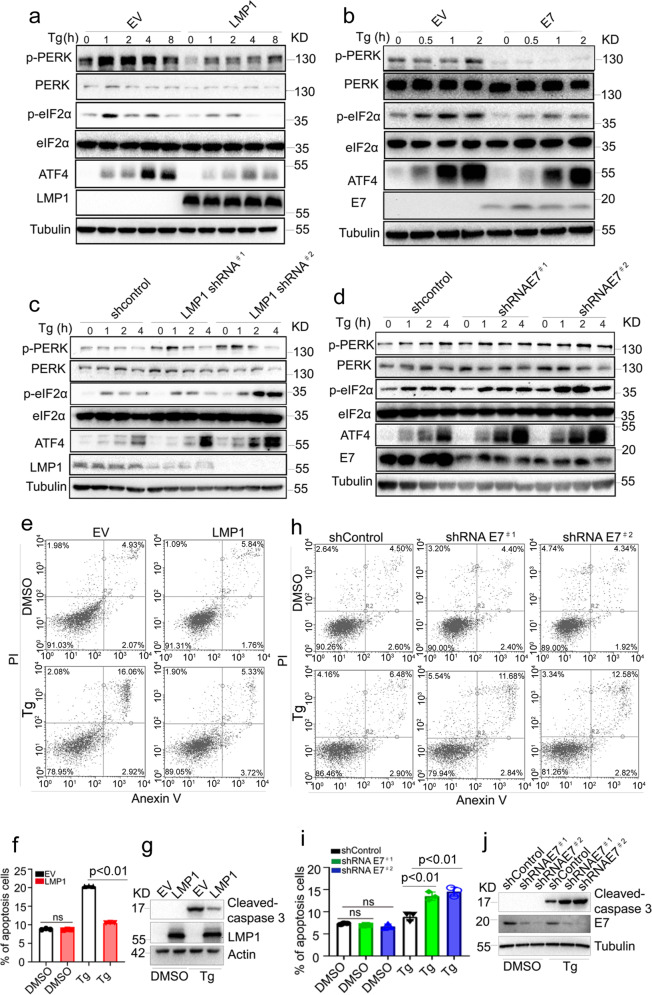
LMP1 and E7 inhibit the PERK-mediated UPR. **a**, **b** CNE1-EV or CNE1-LMP1 (**a**) and C33A-EV or C33A-E7 cells (**b**) were stimulated with 1 μM thapsigargin (Tg) for the indicated time, and the PERK-mediated UPR was evaluated by immunoblotting. **c**, **d** C666-1 (**c**) or HeLa cells (**d**) were transduced with two independent shRNAs targeting LMP1 or E7, followed by 1 μM thapsigargin (Tg) stimulation for the indicated time. The PERK-mediated UPR was examined by immunoblotting. **e** CNE1-EV or CNE1-LMP1 cells were treated with DMSO or Tg (3 μM) for 48 h before harvest. The cells were stained with Annexin V and PI, followed by flow cytometric analysis to assess cell death. The quantification of the apoptotic cells observed (Annexin V^+^) is shown in (**f**). **g** Western blotting was used to analyze cleaved caspase-3 in CNE1-EV and CNE1-LMP1 cells treated with DMSO or Tg for 48 h. **h** HeLa cells transduced with two independent HPV18 E7-specific shRNAs were treated with DMSO or Tg (3 μM) for 48 h before harvest. The cells were stained with Annexin V and PI, followed by flow cytometric analysis to evaluate cell death. The quantification of the apoptotic cells observed (Annexin V^+^) is shown in (**i**). **j** Western blotting was used to analyze cleaved caspase-3 in HeLa cells with or without E7 depletion treated with DMSO or Tg for 48 h. All data are presented as the mean ± SEM of three independent experiments. The values of *p* < 0.05 were considered statistically significant. ns means no significant

Our findings that viral oncoproteins inhibit PERK signaling suggest that viral oncoproteins may inhibit the PERK-mediated UPR in response to treatment with the ER stress inducers. To test this hypothesis, we assessed the kinetics of PERK activation in cells treated with the inducers thapsigargin (Tg) or tunicamycin (Tun). LMP1 or E7 overexpression significantly inhibited Tg- and Tun-induced PERK activation, as shown by decreased levels of p-PERK, p-eIF2a, and the downstream protein ATF4 (Fig. 2a and b and Supplementary Fig. 2a). Depletion of LMP1 or E7 significantly augmented PERK activity over time upon Tg or Tun treatment in C666-1 and HeLa cells, respectively (Fig. 2c and d and Supplementary Fig. 2b–e), supporting that LMP1 and E7 are negative regulators of the PERK-mediated UPR.

Given that the UPR is correlated with apoptosis, we further examined the effects of LMP1 and E7 on cell survival following treatment with Tg or Tun. Overexpression of LMP1 reduced ER stress-induced cell death caused by Tg treatment, as assessed by Annexin V/propidium iodide (PI) staining and analysis of the cleavage of caspase-3 (Fig. 2e–g). In contrast, depletion of E7 enhanced ER stress-induced apoptosis (Fig. 2h–j). Taken together, our results suggest that DNA tumor viruses play vital roles in regulating the PERK-mediated UPR and ER stress.

### Viral oncoproteins physically interact with PERK

**Fig. 3:**
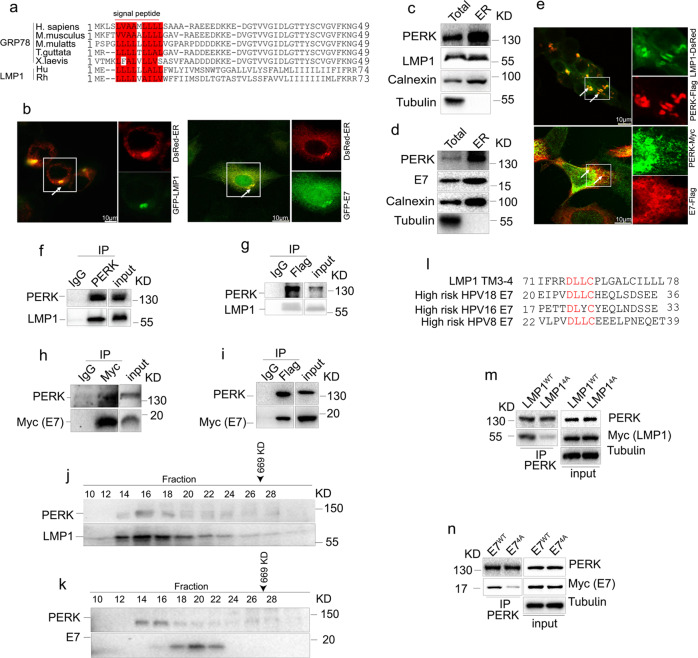
Viral oncoproteins interact with PERK. **a** A sequence alignment of the N-terminal regions of human GRP78, human gammaherpesvirus 4 LMP1 and their sequelogs is shown. Hu: human gammaherpesvirus 4, Rh: Macacine gammaherpesvirus 4. The red box indicates similar residues in mature GRP78 and LMP1. **b** 293T cells were cotransfected with LMP1-GFP or E7-GFP and ER-DsRed for 24 h. Colocalization of LMP1 (left) or E7 (right) with the ER was evaluated by confocal microscopy. **c**, **d** HEK293T cells transfected with LMP1 or E7 were harvested, and then ER microsomes were purified, followed by immunoblotting to detect the indicated proteins. **e** HEK293T cells were cotransfected with LMP1-DsRed and PERK-Flag (top panel). HeLa cells were cotransfected with E7-Flag and PERK-Myc (bottom panel), followed by immunofluorescence staining to show the colocalization of viral oncoproteins and PERK, which are indicated with arrowheads. **f** PERK was immunoprecipitated from CNE1-LMP1 cells, followed by immunoblotting to show the interaction between LMP1 and PERK. **g** HEK293 cells exogenously expressed PERK-Flag and LMP1, and PERK-Flag was immunoprecipitated. The immunocomplexes were analyzed by western blotting with anti-PERK and anti-LMP1 antibodies. **h**, **i** 293T cells were transfected with PERK-Flag and E7-Myc, followed by coimmunoprecipitation with the indicated antibodies to detect the interaction between E7 and PERK. **j**, **k** Whole-cell extracts from CNE1-LMP1 (**j**) or C33A-E7 (**k**) cells were separated by gel filtration, followed by Western blotting. The elution position of a 669-kDa calibration protein (thyroglobulin) is indicated. **l** A sequence alignment of LMP1 TM3-4 and E7 identified a consensus peptide. **m** HEK293T cells were transfected with LMP1^WT^ or LMP1^4A^, followed by immunoprecipitation with an anti-PERK antibody. The immunocomplexes were analyzed by Western blotting. **n** PERK and exogenous Myc-tagged E7^WT^ or E7^4A^ in HEK293T cells were coimmunoprecipitated, and immunoblotting was performed with the indicated antibodies

We next investigated the mechanism by which LMP1 or E7 antagonize PERK signaling. By protein sequence alignment, we noticed that the N-terminal sequence of LMP1 is highly similar to the N-terminal ER signal sequence of GRP78^36^ (Fig. 3a). In addition, it was reported that E7 binds to the transmembrane domain of Sting,^37^ an ER protein,^38^ suggesting that E7 may be located in the ER. Accordingly, we speculated that viral oncoproteins may interact with PERK in the ER to inhibit PERK activity. To verify this hypothesis, we first examined the cellular localization of LMP1 and E7 in cells. Immunofluorescence analysis showed that LMP1 and E7 were predominantly located in the ER (Fig. 3b). In addition, PERK, LMP1, and E7 were shown to be present at high levels in the ER fraction (Fig. 3c and d). Furthermore, the colocalization of LMP1 or E7 with PERK in cells was observed by confocal microscopy (Fig. 3e). The interaction between PERK and LMP1 or E7 was confirmed by co-immunoprecipitation (co-IP) (Fig. 3f–i). To further validate the association of PERK with LMP1 or E7, protein fractionation by gel filtration was carried out. The elution pattern of PERK largely overlapped with those of LMP1 or E7 (Fig. 3j and k), supporting that LMP1 and E7 interact with PERK in vivo.

LMP1 is a transmembrane protein that comprises six transmembrane domains.^39^ To determine which domain interacts with PERK, we constructed various Myc-tagged LMP1 mutants with different deletions, including ΔTM3-6, ΔTM5-6, ΔTM3-4, TM3-4, and ΔTM1-2/5-6 (Supplementary Fig. 3a). Mapping of the interacting domains indicated that TM3-4 but no other transmembrane domains was responsible for the association with PERK (Supplementary Fig. 3b). Consistently, compared to overexpression of wild-type (WT) full-length LMP1, overexpression of LMP1 ΔTM3-4 showed no inhibitory effect on the level of p-PERK, further indicating that LMP1 TM3-4 is critical for the function of LMP1 in the regulation of PERK activity (Supplementary Fig. 3c). Interestingly, by analyzing the amino acid sequences of LMP1 TM3-4 and HPV E7, we identified a single conserved peptide with the sequence Asp-Leu-Leu-Cys (DLLC) (Fig. 3l). The E7 protein contains CR1 and CR2 domains in the N-terminal. The DLLC motif of E7 located in the CR2 domain. Simultaneous substitution of these sites to Ala (the corresponding mutant was termed 4 A) significantly disrupted the interactions of these proteins with PERK (Fig. 3m and n). Collectively, these data show that both LMP1 and E7 interact with PERK via a conserved 4-amino acid peptide.

### Viral oncoprotein-PERK interactions are essential for PERK-eIF2α pathway blockade

**Fig. 4:**
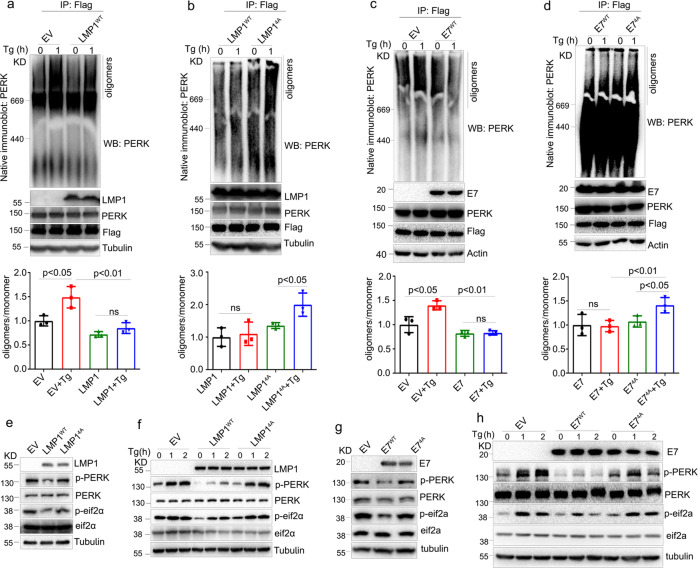
The conserved DLLC peptide in the viral oncoproteins is essential for the PERK-mediated UPR. **a**–**d** HEK293T cells were transfected with PERK-Flag and the indicated viral oncogene plasmids, followed by stimulation with 1 mg/ml Tg for 1 h. PERK was immunoprecipitated, and the oligomerization of PERK was evaluated by negative gel electrophoresis. The quantification of the oligomerization of PERK was shown in the lower panel. **e** CNE1 cells were stably transfected with an empty vector (EV), LMP1^WT^, or LMP1^4A^, followed by immunoblotting to monitor PERK activity. **f** CNE1 cells stably expressing the EV, LMP1^WT^ or LMP1^4A^ were treated with 1 μM Tg at the indicated times. The PERK-mediated UPR was examined by immunoblotting. **g** C33A cells were stably transfected with the EV, E7^WT^ or E7^4A^, followed by immunoblotting to monitor PERK activity. **h** C33A cells stably expressing the EV, E7^WT^, or E7^4A^ were treated with 1 μM Tg at the indicated times. Then, the PERK-mediated UPR was examined by immunoblotting. All data are presented as the mean ± SEM of three independent experiments. The values of *p* < 0.05 were considered statistically significant. ns means no significant

To further investigate how LMP1 and E7 inhibit the PERK-mediated UPR through interactions with PERK, we evaluated the effects of these viral oncoproteins on PERK oligomerization, which has been proposed to be essential for the activation of the UPR.^40^ As expected, the overexpression of LMP1^WT^ or E7^WT^ inhibited oligomerization of PERK (Fig. 4a and c), while enforced expression of mutant LMP1^4A^ or E7^4A^ increased the level of PERK oligomerization compared to their wild types in HEK293T cells treated with Tg (Fig. 4b and d). These results indicated that PERK-viral oncoprotein interactions decreased PERK oligomerization and thereby inhibited PERK activity.

Next, we assessed the effect of PERK-viral oncoprotein interactions on PERK activity and the PERK-mediated UPR. Overexpression of the 4A mutants of LMP1 or E7 had no effect on PERK or eIF2α phosphorylation (Fig. 4e and g). In addition, the levels of p-PERK and p-Eif2a were not reduced by enforced expression of the 4A mutants in comparison with the wild-type controls over time following Tg treatment (Fig. 4f and h). Taken together, these results demonstrate that the interaction with PERK mediated by the conserved DLLC domain of LMP1 and E7 is critical for the inhibition of the PERK-eIF2α pathway.

### DNA tumor virus oncoproteins promote tumor growth by increasing ROS production via inhibiting PERK

**Fig. 5:**
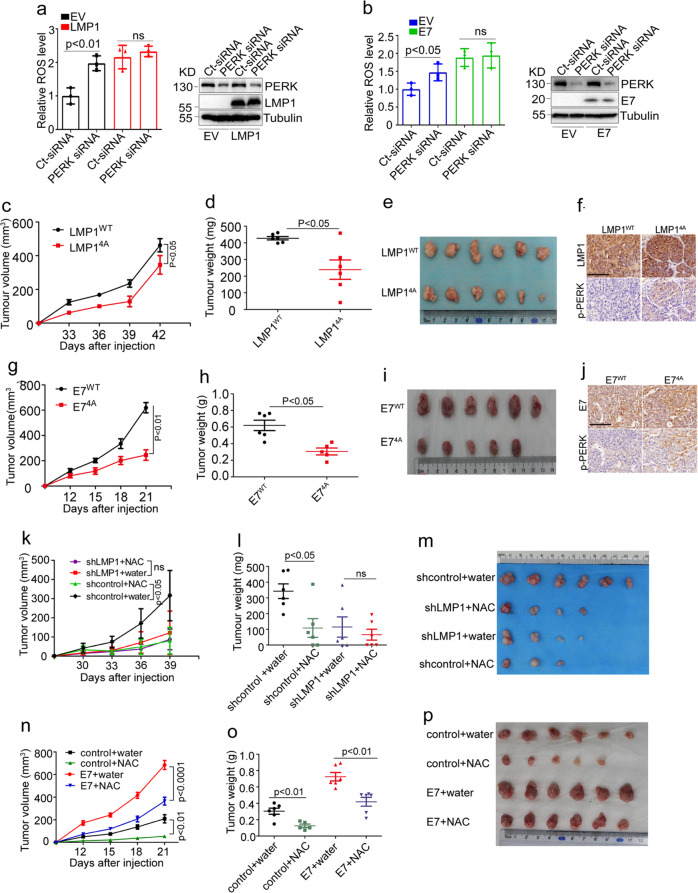
DNA virus oncogenes promote tumor growth by increasing ROS levels. **a** CNE1-EV or CNE1-LMP1 and **b** C33A-EV or C33A-LMP1 cells were transfected with the indicated siRNA, followed by staining with CM-H2DCFDA. Oxidative stress was analyzed by flow cytometry. **c** Tumor growth by 3.5 × 10^6^ subcutaneously injected CNE1 cells stably expressing LMP1^WT^ or LMP1^4A^. **d**, **e** Tumor weight (**d**) and tumor images (**e**) of mice with subcutaneous injection of 3.5 × 10^6^ CNE1 cells stably expressing LMP1^WT^ or LMP1^4A^, at day 42 after implantation. **f** LMP1 and p-PERK immunohistochemical staining of subcutaneous tumors from (**c**). *n* = 6 mice per group, the data are presented as mean ± SEM. **g** Tumor growth by 5 × 10^6^ subcutaneously injected C33A cells stably expressing E7^WT^ or E7^4A^. **h**, **i** Tumor weight (**h**) and tumor images (**i**) of mice with subcutaneous injection of 5 × 10^6^ C33A cells stably expressing E7^WT^ or E7^4A^, at day 21 after implantation. **j** E7 and p-PERK immunohistochemical staining of subcutaneous tumors from (**g**). *n* = 6 mice per group, the data are presented as the mean ± SEM. **k** Tumor growth by 2.5 × 10^6^ subcutaneously injected CNE1-LMP1 cells transduced with the indicated shRNA in response to N-acetylcysteine (NAC) treatment (40 mM in drinking water). **l**, **m** Tumor weight (**l**) and tumor images (**m**) of mice with subcutaneous injection of 2.5 × 10^6^ CNE1-LMP1 cells transduced with indicated shRNA, at day 42 after implantation. **n** Tumor growth by 5 × 10^6^ subcutaneously injected C33A cells stably expressing E7 in response to NAC treatment. **o**, **p** Tumor weight (**o**) and tumor images (**p**) of mice with subcutaneous injection of 5 × 10^6^ C33A cells stably expressing E7, at day 21 after implantation. The values of *p* < 0.05 were considered statistically significant. ns means no significant

PERK specially phosphorylates serine 40 in Nrf2, an important transcription factor involved in antioxidant response, increase cellular antioxidant ability, and thereby eliminate cellular ROS.^41,42^ Given that PERK is an important effector in the maintenance of redox homeostasis, we next examined whether LMP1 and E7 are involved in ROS production by inhibiting PERK activity. Overexpression of viral oncogenes increased cellular ROS levels (Fig. 5a and b). siRNA-mediated depletion of PERK caused a rapid increase in ROS levels in CNE1 and C33A cells. However, no obvious change in ROS levels was observed in the PERK-depleted cells stably transfected with LMP1 in CNE1 or E7 in C33A (Fig. 5a and b), indicating that the viral oncoproteins increased cellular ROS levels by inhibiting PERK activity.

To confirm the hypothesis which viral oncoproteins promote tumor growth by increasing ROS production via inhibiting PERK, we implanted cells stably expressing WT or 4A-mutant LMP1 or E7 into nude mice, and observed a remarkable decrease in tumor growth in the mice implanted with cells stably expressing a 4A-mutant oncogene (Fig. 5c–e and g–i). Compared with the tumors expressing WT LMP1 or E7, tumors expressing 4A-mutant LMP1 or E7 exhibited upregulation of p-PERK level (Fig. 5f and j). Taken together, these results showed that the interactions of viral oncoproteins with PERK contribute to tumorigenesis in vivo by inhibiting PERK activity that causes ROS elevation, at least in part.

An appropriate concentration of ROS is necessary for cell proliferation.^27^ Many studies have shown that elevated ROS levels cause genomic instability and thereby promote tumorigenesis.^23,24^ To examine if LMP1-induced ROS elevation has any effect on tumor cell mutation burden, we performed whole-exome sequencing analysis of the LMP1-expressing cells. Overexpression of LMP1 increased the number of mutations (Supplementary Fig. 4a and 4b), suggesting that LMP1-induced ROS may contribute to the mutation burden in the tumor initiation and progression. We next investigated whether the growth-promoting effect of LMP1 requires ROS. The elevated ROS levels induced by LMP1 could be significantly decreased by treatment with catalase, an enzyme that catalyzes the decomposition of hydrogen peroxide into water and oxygen to protect cells from oxidative damage (Supplementary Fig. 5a). In addition, previous studies showed that LMP1 activated NF-κB/p65 and AKT. Here, we found that ROS clearance resulted in the downregulation of NF-κB/p65 and AKT activity (Supplementary Fig. 5b), which subsequently inhibited LMP1-induced cell growth (Supplementary Fig. 5c). To investigate whether viral oncogene-induced ROS play an important role in tumor progression, CNE1-LMP1 cells with or without LMP1 depletion were subcutaneously injected into nude mice. Consistently, both antioxidant N-acetylcysteine (NAC) treatment and LMP1 knockdown severely attenuated tumor growth in this xenograft model (Fig. 5k–m). Similarly, the E7-induced overgrowth of tumors was also attenuated by NAC treatment in xenograft nude mice (Fig. 5n–p).

PERK has been suggested to function as either a tumor suppressor or a proadaptive tumor promoter depending on the gene dosage. We found that depletion of PERK markedly increased tumor volume and weight in nude mice implanted with LMP1-negative CNE1 cells, and tumor growth was inhibited by NAC treatment (Supplementary Fig. 5d–f). However, PERK knockdown had no obvious effect on tumor growth in mice implanted with CNE1-LMP1 cells. Tumor growth in mice bearing CNE1-LMP1 cells was sensitive to NAC treatment (Supplementary Fig. 5g–i), suggesting that LMP1 promotes tumor progression by the increased ROS level via inhibiting PERK activity.

### Viral oncoprotein expression negatively correlates with PERK phosphorylation in NPC and cervical carcinoma

**Fig. 6:**
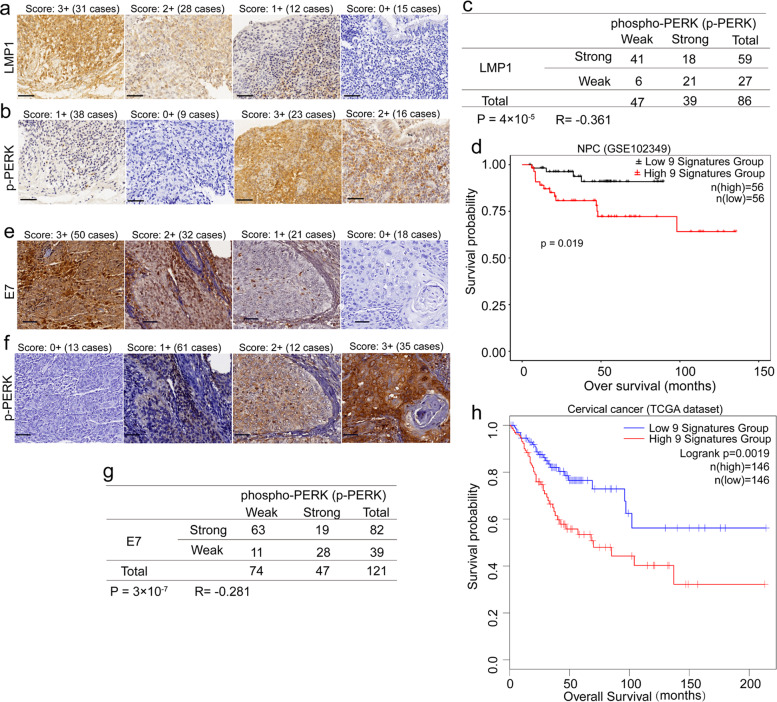
The level of p-PERK is downregulated in viral oncoprotein-positive NPC and cervical cancer specimens. **a**, **b** Representative images of immunohistochemical staining for LMP1 (**a**) and p-PERK (**b**) in LMP1-positive NPC specimens. **c** Inverse correlation between LMP1 expression and the level of p-PERK determined by the *χ*^2^ test. **d** Kaplan–Meier overall survival analysis of a panel of PERK regulated genes in LMP1-positive NPC patients from a nasopharyngeal carcinoma datasets (GSE102349). **e**, **f** Representative staining for E7 (**e**) and p-PERK (**f**) in cervical cancer tissue samples. **g** Inverse correlation between E7 expression and the level of p-PERK determined by the *χ*^2^ test. **h** Kaplan–Meier overall survival analysis of a panel of PERK regulated genes in cervical cancer patients from the TCGA dataset

More than 90% of patients with NPC or cervical cancer are positive for EBV or HPV, respectively.^5,43^ To further confirm the relevance of viral oncoproteins to PERK activity, we subjected clinical tissue samples to immunohistochemistry from 86 patients with primary NPC and 121 patients with cervical carcinoma. The expression levels of LMP1 (Fig. 6a), E7 (Fig. 6e), and p-PERK (Fig. 6b and f) were classified into four levels according to the staining intensity (score: 0 to 3; see the “Materials and methods” section). Analysis of immunohistochemistry on serial tumor sections showed significant inverse correlations between LMP1 and p-PERK (*R* = −0.361; *P* = 4 × 10^−4^) (Fig. 6c) and between E7 and p-PERK (*R* = −0.281; *P* = 3 × 10^−7^) (Fig. 6g) in these NPC and cervical carcinoma tissue samples, respectively. These findings further confirmed the negative regulatory effects of viral oncoproteins on PERK phosphorylation in vivo.

To address the clinicopathological relevance of PERK activity, nine PERK-regulated genes, including GRP78, CHOP, GADD34, ATF4, SLC7A5, SPP1, COL5A2, HIF1A, and COL12A1, were selected as a signature, and tested its association with clinical outcomes of the cancers. As expected, this 9 genes panel showed a negative association with overall survival in both nasopharyngeal carcinoma (Fig. 6d) and cervical carcinoma (Fig. 6h). Previous reports showed that patients with EBV- or HPV-positive cancer had a better prognosis than those with the virus-negative cancer.^5–7^ Present finding may account for the better prognosis of the cancer patients with EBV or HPV infection due to the low PERK activity mediated by DNA tumor virus.

### DNA tumor virus oncogenes inhibit PERK-mediated Nrf2 phosphorylation and antioxidant response

**Fig. 7:**
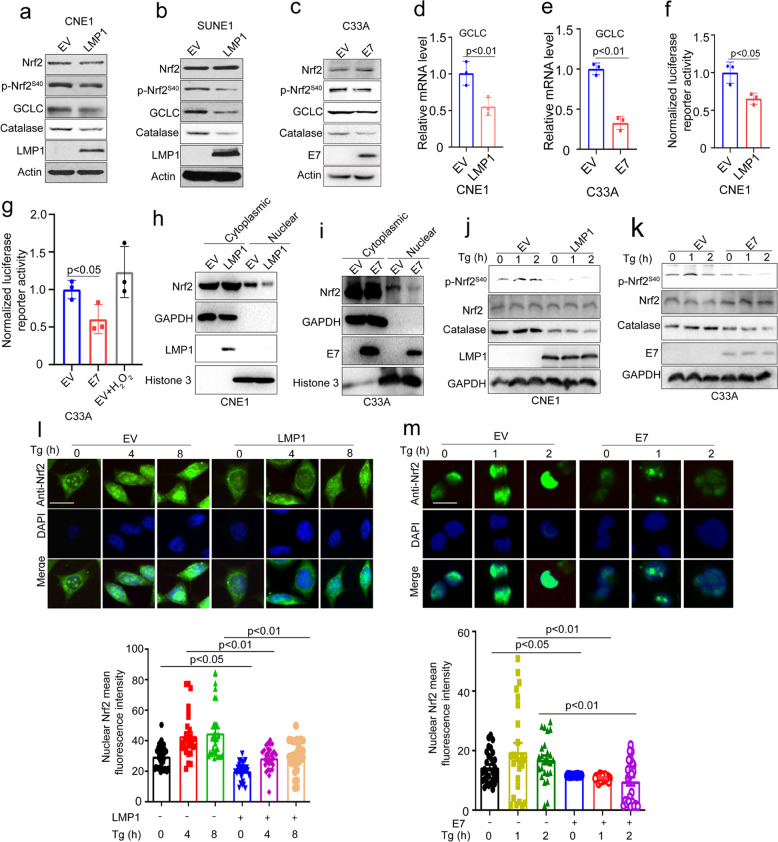
DNA tumor virus oncogenes inhibit PERK-mediated Nrf2 phosphorylation at S40 and antioxidant response. **a**, **b** Immunoblotting of Nrf2, p-Nrf2S40, GCLC, Catalase, LMP1, and Actin in LMP1-transduced CNE1 and SUNE1 cells. **c** Immunoblotting of Nrf2, p-Nrf2S40, GCLC, Catalase, E7, and Actin in E7-transduced C33A cells. **d**, **e** qPCR of GCLC in LMP1-transduced CNE1 and E7-tranduced C33A cells, in (**d**, **e**). **f**, **g** Luciferase assays of Nrf2 activity in LMP1-tranduced CNE1 or E7-tranduced C33A cells transfected with a Nrf2 luciferase reporter. **h**, **i** Immunoblotting of Nrf2 in cytoplasmic and nuclear fractions of LMP1- and E7-transduced CNE1 and C33A cells, respectively. GAPDH and Histone 3 were used as cytoplasmic and nuclear markers, respectively, in (**h**) and (**i**). **j**, **k** Immunoblotting of Nrf2, p-Nrf2^S40^, GCLC, Catalase, Actin and LMP1 or E7 in LMP1-transduced CNE1 and E7-tranduced C33A cells treated with Tg for 1 and 2 h, respectively, in (**j**) and (**k**). **l** LMP1-overexpressed CNE1cells proliferating on class coverslips were treated wih 1.5 µM Tg for the indicated time and fixed, and immunofluorescence analysis was performed with an anti-Nrf2 antibody and DAPI. The quantification data was shown in the lower panel. Data were presented as mean ± SEM (30 cells from 5 randomly selected pictures). Representative images are shown. Scale bars, 10 μm. **m** E7-overexpressed C33A cells proliferating on class coverslips were treated with 1.5 µM Tg for the indicated time and fixed, and immunofluorescence analysis was performed with an anti-Nrf2 antibody and DAPI. The quantification data was shown in the lower panel. Data were presented as mean ± SEM (30 cells from 5 randomly selected pictures). Representative images are shown. Scale bars, 10 μm. The *p* values were determined by two-tailed *t* test. The values of *p* < 0.05 were considered statistically significant. ns means no significant

PERK specially phosphorylates Nrf2 at S40 to increase cellular antioxidant ability, and thereby promote cellular survival.^41^ This, together with our findings that DNA tumor virus oncogenes inhibit PERK activity and increase cellular ROS level, suggests that PERK inhibition by DNA tumor virus oncogenes may lead to suppression of Nrf2 activity. To verify the hypothesis, we determined the phosphorylation level of Nrf2 at S40 in LMP1- or E7-transduced cells. We found the expression of viral oncogenes inhibited phosphorylation of Nrf2 at S40 and downstream antioxidant genes expression (Fig. 7a–e and Supplementary Fig. 6a and b). The Nrf2 luciferase reporter gene assay showed that overexpression of viral oncogenes in CNE1 or C33A cells markedly suppressed the Nrf2 luciferase activity (Fig. 7f and g). H_2_O_2_ treatment was used as a positive control group to test whether the luciferase reporter system working (Fig. 7g). Analysis of protein via nuclear and cytoplasmic extraction demonstrated that viral oncogenes inhibited Nrf2 nuclear localization (Fig. 7h, i and Supplementary Fig. 6c).

To determine whether viral oncogenes can inhibit PERK-mediated Nrf2 phosphorylation, we compared Nrf2 activation in viral oncogenes over-expressed cells to controls. Control cells treated with Tg resulted in robust phosphorylation of Nrf2, whereas viral oncogenes-overexpressing cells failed to response to Tg and activate Nrf2 (Fig. 7j and k). Confocal microscopy further confirmed that viral oncogenes inhibited Nrf2 nuclear localization upon Tg stimulation (Fig. 7l and m). We therefore concluded that viral oncogenes inhibited Nrf2 phosphorylation through inhibiting PERK, and thereby attenuated cellular antioxidant response.

### DNA viral oncoproteins sensitize NPC and cervical cancer cells to paclitaxel treatment in tumor-bearing mice through inhibition of PERK

**Fig. 8:**
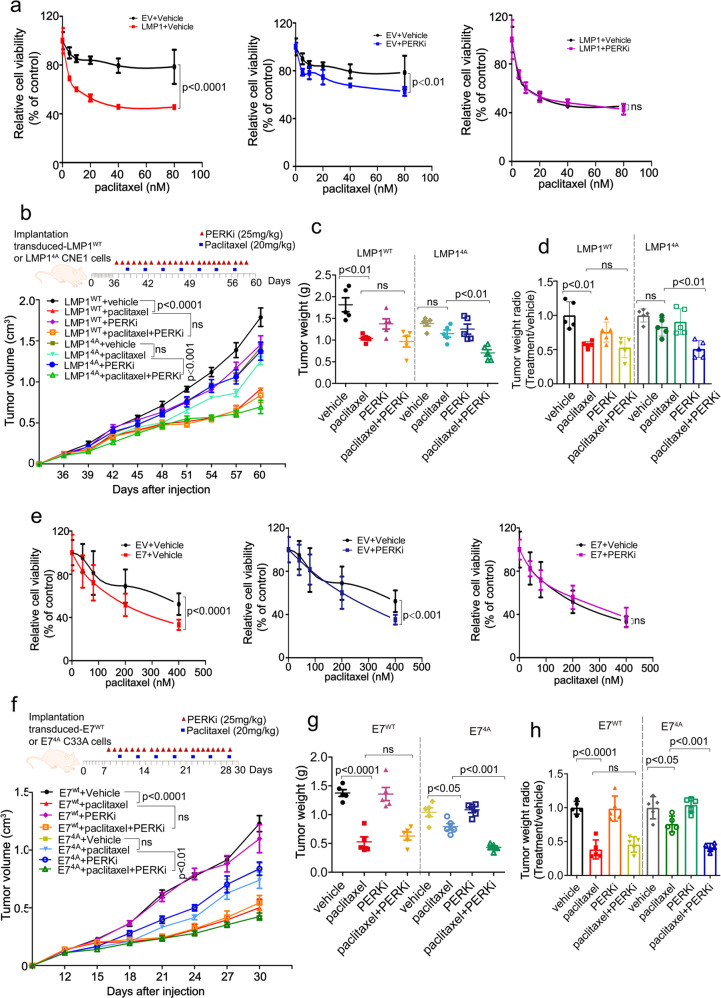
PERK inhibition by DNA tumor virus oncogenes increases cancer cell chemosensitivity. **a**, **e** CNE1-EV or CNE1-LMP1 (**a**) and C33A-EV or C33A-E7 cells (**e**) were pretreated with a vehicle or PERK inhibitor (PERKi) for 24 h, followed by treatment with paclitaxel at the indicated doses for 48 h. Cell viability was measured using a CCK8 assay. Data are presented as the mean ± SEM. **b**–**d** Tumor growth by 3 × 10^6^ subcutaneously injected CNE1 cells stably expressing LMP1^WT^ or LMP1^4A^. **b** CNE1 cells stably expressing LMP1^WT^ or LMP1^4A^ (3 × 10^6^) were injected into nude mice. Once the tumor volume reached 100–120 mm^3^, the mice were treated once daily with a vehicle or PERKi (25 mg/kg) by oral administration and twice weekly with paclitaxel (20 mg/kg) or PBS by intraperitoneal injection according to the schedule described in the “Materials and methods”. The tumors were collected on day 60 and tumor weight (**c**) was quantified. The raw data are shown in (**c**), and normalized data are shown in (**d**). **f**–**h** A total of 3 × 10^6^ C33A cells stably expressing E7^WT^ or 6 × 10^6^ C33A cells stably transfected with E7^4A^ were transplanted into nude mice (**f**). Once the tumor volume reached 60–80 mm^3^. The mice were treated as described above, except the tumors were collected on day 30. Tumor weight was quantified and is shown in (**g**); normalized data are shown in (**h**). *n* = 5 mice per group, the data are presented as the mean ± SEM. The values of *p* < 0.05 were considered statistically significant. ns means no significant

To further investigate the mechanism underlying the improved prognoses of patients with EBV- or HPV-positive cancers, we examined the effects of viral oncoproteins on cancer cell sensitivity to chemotherapeutic drugs. Partial inhibition of the proapoptotic UPR mediated by PERK contributes to tumorigenesis and tumor progression.^13,15,16^ However, PERK inhibition has also been reported to sensitize cancer cells to chemotherapeutic drugs.^21,44,45^ Since paclitaxel and 5-FU are the first-line chemotherapeutic drugs for NPC and cervical cancer patients,^46,47^ we tested if DNA tumor virus could chemo-sensitize cancer cells via inhibition of PERK activity, thereby leading to a favorite prognosis. Indeed, PERK inhibition or overexpression of LMP1 or E7 markedly increased the sensitivity of cancer cells to paclitaxel (Fig. 8a and e). However, pretreatment with a PERK inhibitor could not further sensitize cells stably expressing LMP1 or E7 to paclitaxel (Fig. 8a and e). The level of PERK phosphorylation in response to the PERK inhibitor and/or paclitaxel in the presence or absence of LMP1 or E7 was consistent with cell viability (Supplementary Fig. 7a and b).

We further investigated the effect of DNA tumor virus oncoproteins on paclitaxel treatment efficacy in xenograft mouse models, in which paclitaxel was given twice weekly by intraperitoneal route and/or PERK inhibitor was administrated intragastrically once daily. Consistently, paclitaxel treatment markedly inhibited the growth of tumors stably expressing wild type viral oncogenes but exerted no significantly inhibitory effect on tumors stably expressing the 4A-mutant viral oncogenes (Fig. 8b–d and f–h, Supplementary Fig. 7c and d). PERK inhibitor treatment alone showed no significant effect on tumor growth. However, combinational treatment of paclitaxel and PERK inhibitor could further sensitize the tumor of the 4A-mutant cells, but had less effect on the tumor from cells expressing wild-type viral oncogenes (Fig. 8b–d and f–h, Supplementary Figure 7c and d). Taken together, these results demonstrate that the DNA tumor virus oncogenes LMP1 and HPV E7 sensitize NPC and cervical cancer cells to paclitaxel treatment through inhibition of PERK.

## Discussion

Oncogenic DNA viruses have been implicated in oncogenesis and development of many tumors. However, it remains unclear why the DNA virus positivity is associated with a favorite prognosis for cancer patients. Here, we found that the DNA virus oncoproteins LMP1 and E7 inhibit PERK activity through a conserved DLLC domain, which on the one hand results in tumor progression driven by ROS signaling, but on the other hand, sensitize tumors to chemotherapy possibly via disturbed redox homeostasis.

Functional cooperation between PERK and LMP1 is controversial. Previous reports indicated that high expression of LMP1 in Burkitt lymphoma (BL) cells results in activation of PERK and PERK-mediated UPR.^48^ These discrepancies from the present report may be attributed to the following reasons. Virologically, EBV presence in BL and NPC is in different types of latencies, where types I or III are in BL, and type II in NPC. The major difference of molecular characteristics between the latency II and III is presence (type III) and absence (type II) of EBNA-2 expression.^49^ At transcriptional level, EBNA-2 could transactivates c-myc expression through an EBNA-2 responsive element in the c-myc promoter.^50^ The dysregulation of c-myc oncogene triggers the UPR as an adaptive strategy for BL proliferation.^51^ In addition, in B lymphoma cells, Ig is produced at a consistent high level that causes UPR.^52,53^ Thus, EBV infection could enhance UPR in BL cells either via EBNA2 transactivation of c-myc, or through LMP1 to increase Ig production that further augments UPR.^48,54^

PERK, an eIF2α kinase, play an important role in host defense. eIF2α, a critical translation initiation factor, is phosphorylated and inactivated by eIF2α kinase thus translation is inhibited to thwart the production of viral polypeptides.^55,56^ Thus, eIF2α phosphorylation and its kinase are regarded as sentinel host defense molecules that hold infection and hastily draw the viral lifecycle to an unnatural end.^56^ Many viruses encode antagonists to inhibit PERK pathway for a productive infection. For example, HSV-1-encoded ICP34.5 and Chikungunya-encoded nsP4 suppress eIF2α phosphorylation and thereby translation attenuation does not occur.^57,58^ Our results suggest the inhibition of PERK by LMP1 or E7 may facilitate establishment of persistent infection of EBV or HPV in epithelial cells.

In this report, we demonstrated that the viral oncogenes-mediated inhibition of PERK led to a high level of ROS, accelerating tumor progression. Indeed, as the PERK-mediated UPR induces either survival or apoptosis in response to ER stress, it may facilitate or suppress malignant transformation depending on the context. For example, although deletion of PERK attenuates Neu-dependent mammary tumor progression and lung metastasis, long-term PERK inactivation promotes spontaneous mammary tumorigenesis owing to increased genomic instability.^59^ PERK activation also contributes to MYC-induced cell transformation and tumorigenesis through autophagy.^60^ This may be related to PERK-mediated eIF2α phosphorylation, resulting in increases of the levels of ATF4, CHOP, and factors that activate the transcription of many autophagy genes. However, it was reported that induction of CHOP in response to chronic ER stress promoted cell apoptosis to prevent tumorigenesis. In this study, LMP-1 caused downregulation of PERK-mediated UPR genes accompanied the upregulation of IRE1α-mediated UPR genes. Our explanation for this is that LMP-1 could impact on UPR responses via suppression of PERK activity in one hand, and activation of the genes downstream of IRE1α pathway through different transcriptional factors, but not XBP1, in other hand. Indeed, many genes downstream of IRE1α-XBP1 pathway are not only regulated by XBP1, but also regulated by other transcription factors such as HIF-1α and NF-κB.^61–63^ Several genes listed in our figure (Supplementary Fig. 1d) were able to be regulated by HIF-1α and NF-κB, such as SHC1,^64^ HSPA5^65^, and HDGF.^66^ Furthermore, both NF-κB activity^67^ and HIF-1α^68^ can be activated by LMP1 through ROS. Thus, LMP1 may upregulated the IRE1α-XBP1 targeted UPR genes through non-XBP1 transcriptional regulation without activating IRE1α, such as HIF-1α and NF-κB. In this way, the virally infected cells could not only degrade the excessive proteins from viral replication, but also avoid apoptosis caused by IRE1α activation.^9^ Taken together, these results showed LMP1 inhibits PERK-mediated UPR pathways.

Several studies have reported that patients with EBV-positive tumors, including gastric adenocarcinomas, cHL, and HPV-positive cervical cancer, have a better prognosis than patients with EBV- or HPV-negative tumors. In addition, although the plasma EBV DNA level is associated with survival in NPC, NPC patients with a high EBV DNA level have better prognosis for distant metastatic recurrence than those with a low EBV level or without EBV infection.^69,70^ Some of EBV positive BL and EBV associated gastric cancer (EBVaGC) that comprises ~10% of gastric carcinomas usually do not express LMP1, but show a better prognosis. We reckon that although this portion of the cancers do not express LMP-1, but express some other important viral oncogenes, the function of which remains to be clarified. However, it has been suggested that the prognostic factors for EBV-associated tumors may include age, EBV gene expression, tumor mutation burden and immune infiltration.^71,72^ Particularly, EBV was found to be more frequent in the MSS/TP53 + subtype of EBVaGC, with significant enrichment of PIK3CA and ARID1A mutations, and increased immune infiltrates.^73^ In this study, we showed that DNA tumor viruses could markedly promote tumor progression by inhibiting PERK activity. Furthermore, we showed that DNA tumor virus infection could also sensitize cancer cells to chemotherapy by increasing PERK-mediated ROS production. Indeed, UPR inhibition and increased cellular ROS levels could sensitize cancer cells to chemotherapy as the UPR promotes adaptation and drug resistance.^20,74^ Therefore, our study, for the first time, provides an explanation for the long-standing question of why HPV- or EBV-positive patients show a relatively good prognosis.

Taken together, our findings show that PERK inhibition by DNA tumor virus oncoproteins promotes tumor progression but this inhibition also contributes to the improved response of cancer patients to chemotherapy. This is highly relevant in NPC and cervical cancers, as HPV- or EBV-positive patients show a relatively good prognosis. Targeting the PERK pathway may provide therapeutic benefits to cancer patients.

## Materials and methods

### Antibodies and reagents

Antibodies against tubulin (mouse polyclonal, 1:5000), actin (mouse polyclonal, 1:5000), PERK (bovine monoclonal, 1:500, sc-9481), p-PERK (rabbit polyclonal, 1:1000, sc-32577), cyclin D1 (mouse polyclonal, 1:500, sc-20044), and HPV E7 (rabbit polyclonal, 1:1000) were from Santa Cruz. Antibodies against Eif2a (rabbit polyclonal, 1:1000, 5324), p-Eif2a (rabbit polyclonal, 1:1000, 9721), Phospho-Akt (Thr308) (1:1000, 13038), Phospho-NF-κB p65 (Ser536) (1:1000, 3033), ATF4 (rabbit polyclonal, 1:1000, 11815), CHOP (rabbit polyclonal, 1:1000, 5554), cleaved-caspase 3 (rabbit polyclonal, 1:1000, 9661), and cleaved-PARP (rabbit polyclonal, 1:1000, 5625) were from Cell Signaling Technology. Anti-Ki67 (rabbit polyclonal, 1:2000, Ab15580) antibody were purchased from Abcam. Anti-LMP1 (mouse monoclonal, 1:200, M0897) antibody was from DAKO. Anti-Myc (rabbit polyclonal, 1:1000, PAB12716) antibody was from Abnova. Anti-FLAG (mouse monoclonal, M2, 1:1000, F3165) antibody was from Sigma-Aldrich. The PERK inhibitor (GSK2606414) was purchased from Selleck. Other chemicals, as well as Endoplasmic Reticulum isolation kit (ER0100) were purchased from Sigma-Aldrich.

#### EBV infection

A recombinant EBV carrying a neomycin resistance (neo^r^) gene and a green fluorescent protein (EGFP) gene was obtained from Zeng Musheng Lab. Infectious rEBV was obtained from medium of the EGFP-neo^r^ EBV-infected Akata cells in which EBV production had been induced by surface immunoglobulin G (sIgG) cross-linking as previous described.^75,76^

#### Cell culture

Human cervical cancer cell lines (C33A, HCC94, Hela, HT3, Caski, and Siha) and HEK293T cells were purchased from American Type Culture Collection. Human EBV-negative NPC cell lines CNE1, HONE1, HNE2, CNE2, SUNE1, and EBV-positive NPC cell line C666-1^77^ were obtained from Institute of Cancer Research, Central Southern University. All of human cervical cancer cell lines and HEK293T cells were cultured in DMEM (Gibico) medium and supplemented with 10% fetal bovine serum (FBS; Gibico, Australia) and 100 mg per ml ampicillin/streptomycin (Hyclone). All of human nasopharyngeal carcinoma cell lines were cultured in RPMI1640 (Gibico) medium and supplemented with 10% fetal bovine serum and 100 mg per ml ampicillin/streptomycin. All the cell lines were mycoplasma negative and authenticated by STR profiling. All of cell lines were maintained at 37 °C with 5% CO_2_.

### Expression constructs and transfection

E7 construct was cloned into pcDNA3.1-myc-tag or pcDNA3.1-Flag-tag or pKC-Myc-tag vector by using MSCV-C E7 (Addgene, 37886) as a template. Constructs for LMP1 and its mutants construct was cloned into pcDNA3.1-myc-tag or pcDNA3.1-Flag-tag or pKC-Myc-tag vector by using pSG5-LMP1 as a template (provided by Cao Ya). LMP1 encoding sequence was amplified by PCR and cloned in the acceptor vector by using BamHI and EcoRI restriction sites. PERK construct were cloned into pcDNA3.1-flag-tag vector by using pCMV6-PERK (OriGene) as a template. In addition, wild-type LMP1, E7 (pLV- E7^WT^), as well as their mutants were cloned into lentiviral vectors. Plasmid transfection was performed with viafect (Promega), according to the manufacturer’s instructions.

#### RNA-seq assay and data analysis

A minimum of 3 μg of total RNA was isolated, and cDNA libraries were prepared using the Illumina TruSeq mRNA stranded library prep kit (Illumina) according to the manufacturer’s protocol. The cDNA library was prepared for sequencing on an Illumina HiSeq 2000 sequencing platform (BerryGenomics). Reads per kilobase pair per million reads mapped (RPKM) value for each gene were estimated. A gene is considered significantly differentially expressed if its expression differs between any two samples with the fold change >2 and the *p* value <0.05 as calculated by Cufflinks. For analyzing public data set (GSE102349), the patients were divided into two groups according to the median expression level of all EBV encoded genes. And then the data was analyzed by the online tool iDEP (http://bioinformatics.sdstate.edu/idep/).

#### RNA interference

Individual siRNAs against PERK, E6, E7, L1, L2, and E5 (Genepharma) were introduced into cells using Dharmafect (Thermo Scientific), according to the manufacturer’s instructions. All target sequences are shown in Table S1.

#### Lentiviral shRNA cloning, production, and infection

To generate PERK-, LMP1-, and E7-knockdown cells, oligonucleotides were cloned into pLVX by using BamHI and EcoRI restriction sites. Lentiviral packaging plasmids psPAX2 and pMD2.G were co-transfected with the backbone plasmid into HEK293T cells for virus production. Two days after transfection, medium containing virus particles were used to infect mammalian cells. All infected cells were selected in 1.0 μg/ml puromycin in culture medium. The oligonucleotide pairs used were as follows: LMP1 #1, (5′-ATCCGGAATTTGCACGGACAGGC-3′) and #2, (5′-ATCCGCTCTCTATCTACAACAAA-3′); E7 #1, (5′-ATCCGAAAACGATGAAATAGATG-3′) and #2, (5′-ATCCGCATTTACCAGCCCGACGAG-3′); PERK #1, (5′-ATCCGCCTCAAGCCATCCAACATATT-3′), #2, (5′-ATCCGGAAACAGCTA-3′), and #3, (5′-ATCCGGGAACGACCTGAAGCTATAAA-3′).

#### Immunoblotting

The cultured cells were rinsed once with ice-cold PBS and then were lysed in lysis buffer (150 mM NaCl, 25 mM Tris, pH 7.6, 1 mM EDTA, 1% NP-40, 1% sodium deoxycholate, 0.1% SDS) with protease inhibitors (Roche) and phosphatase inhibitors (Millipore). Cell lysates were centrifuged at 14,000 × *g* for 15 min, and the protein concentrations were determined by BCA kit (Thermo Scientific). Equivalent protein quantities were subjected to SDS-PAGE, and transferred to PVDF membranes (Millipore). Membranes were blocked with 5% non-fat milk or 5% BSA for 1 h at room temperature and then probed with the indicated primary antibodies, followed by the appropriate HRP-conjugated anti-mouse/rabbit or anti-goat (Santa Cruz) secondary antibodies for 1 h at room temperature. Immunoreactive bands were visualized with Bio-Rad system.

#### Immunoprecipitation

Cells were collected and lysed in 0.5 ml lysis buffer (25 mM Tris-HCl pH 7.4, 150 mM NaCl, 1% NP-40, 1 mM EDTA, 5% glycerol) with protease inhibitors (Roche). The lysates were immunoprecipitated with 2 μg specific antibody overnight at 4 °C, followed by incubating with 30 μl A/G agarose beads (Santa Cruz, SC-2003) for another 3 h. Thereafter, the precipitants were washed five times with lysis buffer, and the immune complexes were boiled with loading buffer for 5 min and analyzed by SDS-PAGE at room temperature. The following antibodies were used for immunoprecipitation: anti-PERK (Santa Cruz, sc-9481), anti-Flag (Sigma, F1804, clone M2), anti-Myc (Abnova, PAB12716).

#### Gel filtration

CNE1-LMP1 or C33A-E7 cells were lysed in Tris-Hcl buffer (Tris-HCl 25 mM pH 8.0, 250 mM NaCl). The suspension was sonicated and centrifuged at 12,000 × *g* for 15 min. Whole-cell extracts were applied to a Superose 610/300 GL column for gel filtration. Aliquots of each fraction were subjected to SDS–PAGE. The PERK, LMP1, and E7 content were measured by immunoblotting.

#### Cell proliferation assay

Cell proliferation rates were determined as described. Briefly, cells were seeded in 6-well plate and cultured in 2 ml of medium supplemented with 10% FBS containing catalase. Cell number at the indicated time points was determined by counting using automated cell counter.

#### Dual-luciferase reporter assay

Cells of 80% confluence in 6-well plates were transfected using viafect (Promega). The firefly luciferase reporter gene construct (0.5 μg) and the pRL-SV40 *Renilla* luciferase construct (10 ng) were used for co-transfection. After 24–48 h, cell extracts were prepared and the luciferase activity was measured using Dual-luciferase reporter Assay System (Promega).

#### ROS assay

The level of intracellular ROS was evaluated by staining the cells with carboxy-H2DCFDA (Invitrogen). Briefly, cells were incubated with 10 mM carboxy-H2DCFDA for 30 min at 37 °C before fluorescence measurements. Cells were washed and analyzed by flow cytometry (Millipore).

#### Flow cytometry analysis

Cells were treated with Tg for 72 h. After washing with cold PBS, all cells including both floating and attached cells were collected by tripsin digestion and re-suspended in PBS, followed by double stained with Annexin V-FITC and propidium iodide. Five thousand cells were analyzed by flow cytometry for apoptotic cells. Data were analyzed from three independent experiments and are shown as the average mean ± SEM.

#### Endoplasmic reticulum isolation

ER microsomes were isolated using ER isolation kit (Sigma) according to manufacturer’s protocol.

#### Cell viability assay

Relative cell viabilities were determined using CCK8 kit (Promega). Relative cell viability assay was performed using a microplate reader (Perk Elmer).

#### Native gel analysis

Negative gel analysis was performed as described.^78^ Briefly, cells were lysed using 1% NP40 lysis buffer (20 nM Tris-HCl pH 7.5, 150 mM NaCl, 1 mM EDTA, 1% NP40, 1 mM protease) and immunoprecipitated with indicated antibody. For the native elution, the immunocomplexes were eluted with pre-cold 0.1 M glycine (pH 2.5) for 15 min at 4 °C, followed by neutralization with 1.5 M Tris-HCl (pH 8.8). Tris-Glycine-Native sample buffer was added, and samples were immediately analyzed by Native PAGE Gel.

#### In vivo tumorigenesis study

All animal experiments were performed according to a protocol approved by the Institutional Animal Care and Use Committee of Xiangya Hospital. Five to six female 4–6-week-old BALB/c nude mice were randomly assigned to different groups to ensure it meet the criteria for statistical analysis. For tumorigenesis study in a xenograft model, cancer cells were injected subcutaneously into nude mice. Nude mice were treated with 40 mM N-acetylcysteine as described.^23^ Tumor size was measured every three days using a vernier caliper at room temperature, and tumor volume was calculated using the standard formula: 0:5 × *L* × *W*^2^, where *L* is the longest diameter and *W* is the shortest diameter. Mice were euthanized when they met the institutional euthanasia criteria for tumor size and overall health condition. The tumors were removed, photographed, and weighed. A laboratory technician (LY Liu) responsible for animal care and measurement of tumor growth was blinded to the group allocation during all animal experiments and outcome assessment. The animals were weighed, and block randomized according to tumor size into treatment groups of 5 or 6 mice each.

For efficacy studies of PERK inhibitor in combination with paclitaxel in tumor-bearing mice, exponentially growing CNE1 cells with LMP1^WT^ or LMP1^DLLC^ overexpression were implanted subcutaneously into the right flank of 4- to 6-week-old female nude mice. When the tumors reached ~100–120 mm^3^ in size, the animals were weighed, and block randomized according to tumor size into treatment groups of 5 mice each. Mice were treated once daily with GSK2656157 (25 mg/kg) or formulating vehicle by oral administration and twice weekly with paclitaxel (20 mg/kg) or PBS by intraperitoneal injection. Mice were weighed and tumors size was measured by calipers every 3 days. Tumor volume was calculated using the standard formula: 0:5 × *L* × *W*^2^, where *L* is the longest diameter and *W* is the shortest diameter. The nude mice were excluded if the transplanted tumor developed ulceration.

#### Patient study and immunohistochemistry

The NPC and cervical carcinoma tissue were received from Xiangya Hospital. These samples were deparanized and rehydrated. Antigen was retrieved using 0.01 M sodium-citrate buffer (pH 6.0) heated for 20 min at 95 °C. The samples were pretreated with 5% goat serum for 1 h to block antibody nonspecific binding and then incubated with the indicated antibodies at 4 °C overnight. The samples were treated with 0.3% H_2_O_2_ for 15 min to block endogenous peroxidase activity and then incubated with HRP-conjugated secondary antibody for 1 h at room temperature. The following antibodies were used for immunohistochemistry: antibodies against p-PERK (1:200, Santa Cruz), LMP1 (1:200, DAKO), HPV18 E7 (1:100, Santa Cruz), Immunoreactive signal was visualized with a DAB Substrate Kit (ZSGB-BIO). Positive cells were calculated as the number of immunopositive cells × 100% divided by a total numbers/field in 10 random fields. The IHC scoring was reviewd by two pathologists in a double-blind manner. Protein expression levels of all the samples were scored as four grades (0–3), according to the percentage of immunopositive cells and immunostaining intensity. Grade 0–3 represent: 0 (no expression), 1 (low expression), 2 (moderate expression), 3 (high expression). Grade 0 and 1 were defined as low expression and Grade 2 and 3 were defined as high expression. The *χ*^2^ test was used for analysis of the significance of LMP1 or E7 and p-PERK with tumor and the correlation between LMP1 or E7 and p-PERK.

### Statistics

Generally, we followed previous literatures, which represent the field of tumor research, to determine the sample size and the number of experiments [23–25]. GraphPad Prism 8 for Windows (GraphPad Software) was used for all statistical analyses. Statistical analyses were performed with an unpaired, two-tailed Student’s *t* test to compare mean differences between the control and treatment groups. All data shown represent the results obtained from three (or as indicated) independent experiments with standard errors of the mean (mean ± SEM). The variance similar between the groups that are being statistically compared. The *P* value of <0.05 was considered statistically significant. All quantitative data are presented as the mean ± SEM.

## Supplementary information

Supplementary information

Supplementary Table 2

## Data Availability

The gene expression array data have been deposited in the Gene Expression Omnibus database (accession number GSE171664). The rest of datasets used and analyzed during the current study are available from Gene Expression Omnibus database (GSE102349, GSE6791, and TCGA cervical cancer dataset).
