# Supplementary material for: Paradoxical effects of DNA tumor virus oncogenes on epithelium-derived tumor cell fate during tumor progression and chemotherapy response
Source: Signal Transduct Target Ther. 2021 Nov 26;6:408. doi: 10.1038/s41392-021-00787-x (PMC8626493; doi:10.1038/s41392-021-00787-x)
Supplement: Supplementary file 1 — Supplementary information [file 41392_2021_787_MOESM1_ESM.doc]

Supplementary Materials for

Paradoxical effects of DNA tumor virus oncogenes on epithelium-derived tumor cell fate during tumor progression and chemotherapy response

Jiang He^1,2^, Liyu Liu^1,2^，Feiyu Tang^1,2^, You Zhou^3^, Huan Liu^1^, Can Lu^4^, Deyun Feng^4^, Hong Zhu^1^, Yitao Mao^5^, Zhi Li^1,2,6^, Lu Zhang^1,2^, Yuemei Duan^4^, Zhi Xiao^7^, Musheng Zeng^8^, Liang Weng^1,2,9*^, Lun-Quan Sun^1,2, 6,9,10*^

Correspondence to LQS (lunquansun@csu.edu.cn) and LW ([wengliang@csu.edu.cn](mailto:wengliang@csu.edu.cn) )

**This PDF file includes:**

Figures. S1 to S7

Table S1

**
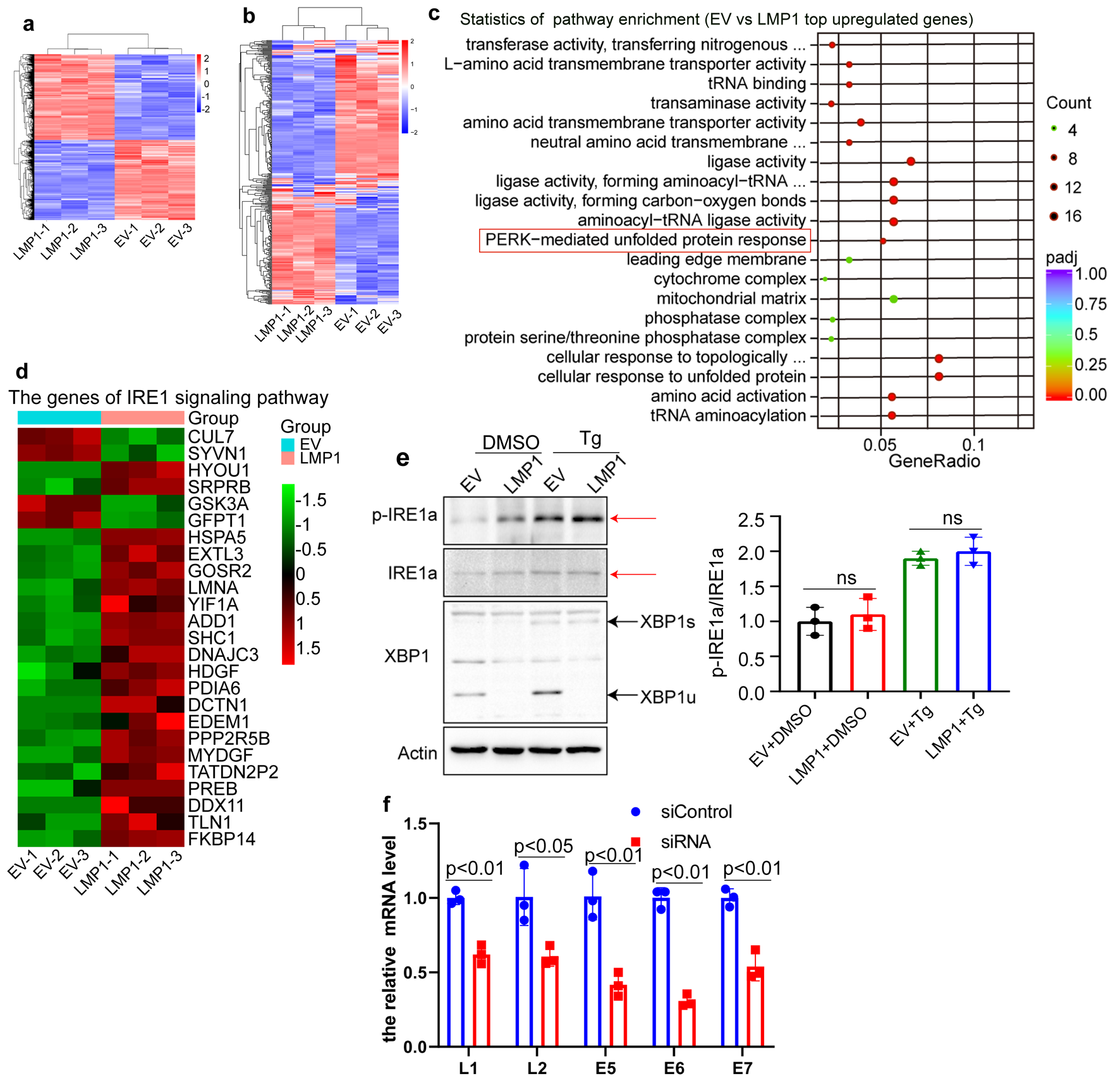
**

**Supplementary Fig. 1. LMP1 downregulates the activity of PERK.** (**a**) Heat map comparing the expression of genes between CNE1-EV and CNE1-LMP1 cells. n=3 independent replicates. (**b**) Heat map of known PERK target genes expressed in CNE1-LMP1 and CNE1-EV cells. n=3 independent replicates. (**c**) PERK downstream genes were enriched by GO. (**d**) Heat maps generated from RNA-seq data (from RNA-seq FPKM values) showing LMP1 upregulated expression of the genes of IRE-XBP1 signaling pathway. n=3 independent replicates. (**e**) CNE-1 transfected with the indicated plasmids and treated with ER stress inducer thapsigargin (Tg), and then evaluated by immunoblotting to examine IRE1a activity. The quantification of IRE activation was measured by the ratio of pIRE/IRE. (f) the knockdown efficiency of HPV genes (L1, L2, E5, E6, E7) is evaluated by Realtime PCR.


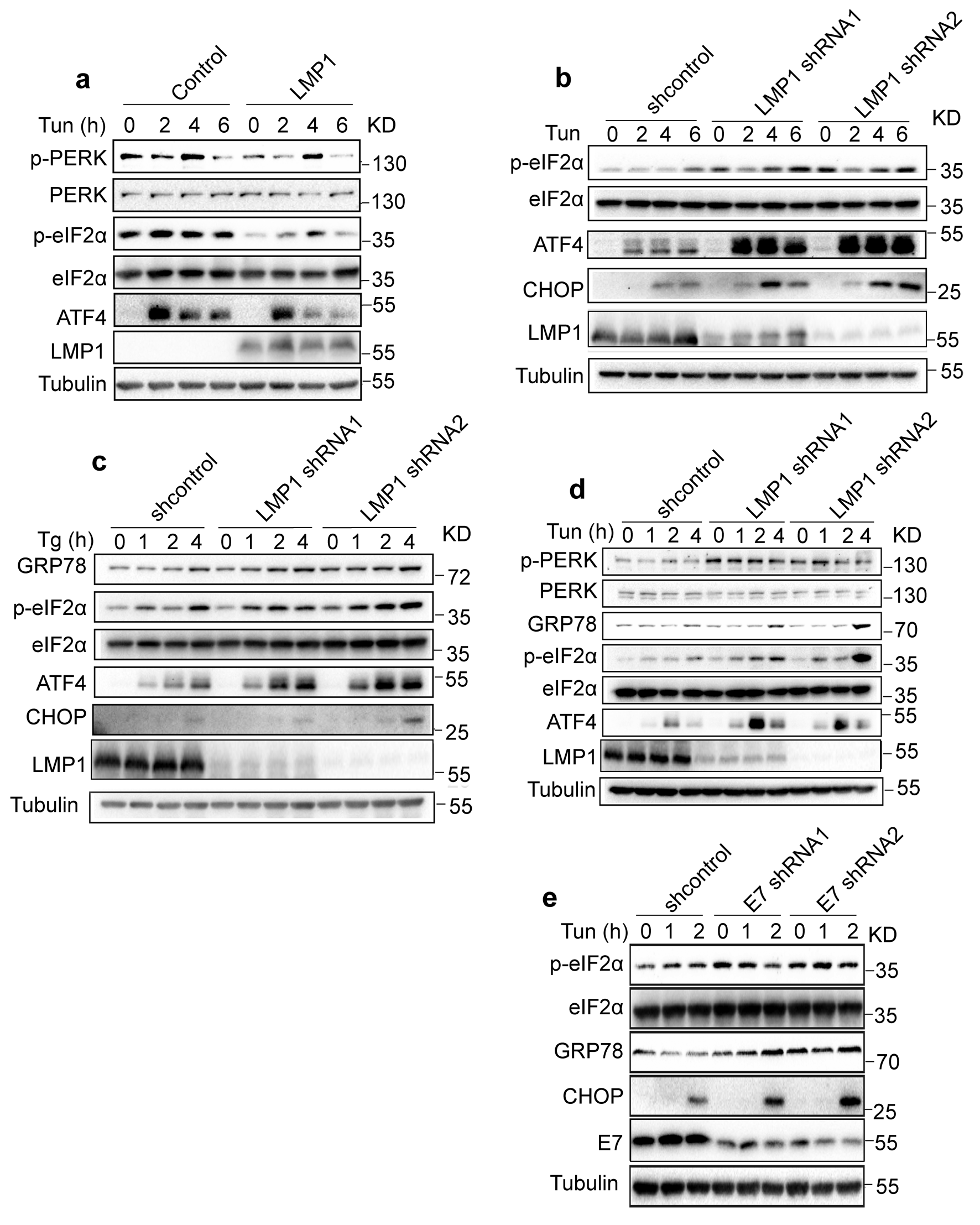


**Supplementary Fig. 2. DNA tumor virus oncogenes modulate the PERK-mediated UPR.** (**a**) CNE1-EV or CNE1-LMP1 cells were stimulated with 1 μM thapsigargin (Tun) for the indicated time, and the PERK-mediated UPR was checked by immunoblotting. (**b**, **c**) CNE1-LMP1 cells were transduced with two independent shRNAs targeting LMP1, followed by 1 μM Tun (**b**) or Tg (**c**) stimulation for the indicated time. The PERK-mediated UPR was examined by immunoblotting. (**d**) C666-1 cells were transduced with two independent shRNAs targeting LMP1, followed by 1 μM Tun stimulation for the indicated time. The PERK-mediated UPR was examined by immunoblotting. (**e**) HeLa cells were transduced with two independent shRNAs targeting E7, followed by 1 μM Tun stimulation for the indicated time. The PERK-mediated UPR was examined by immunoblotting.

**
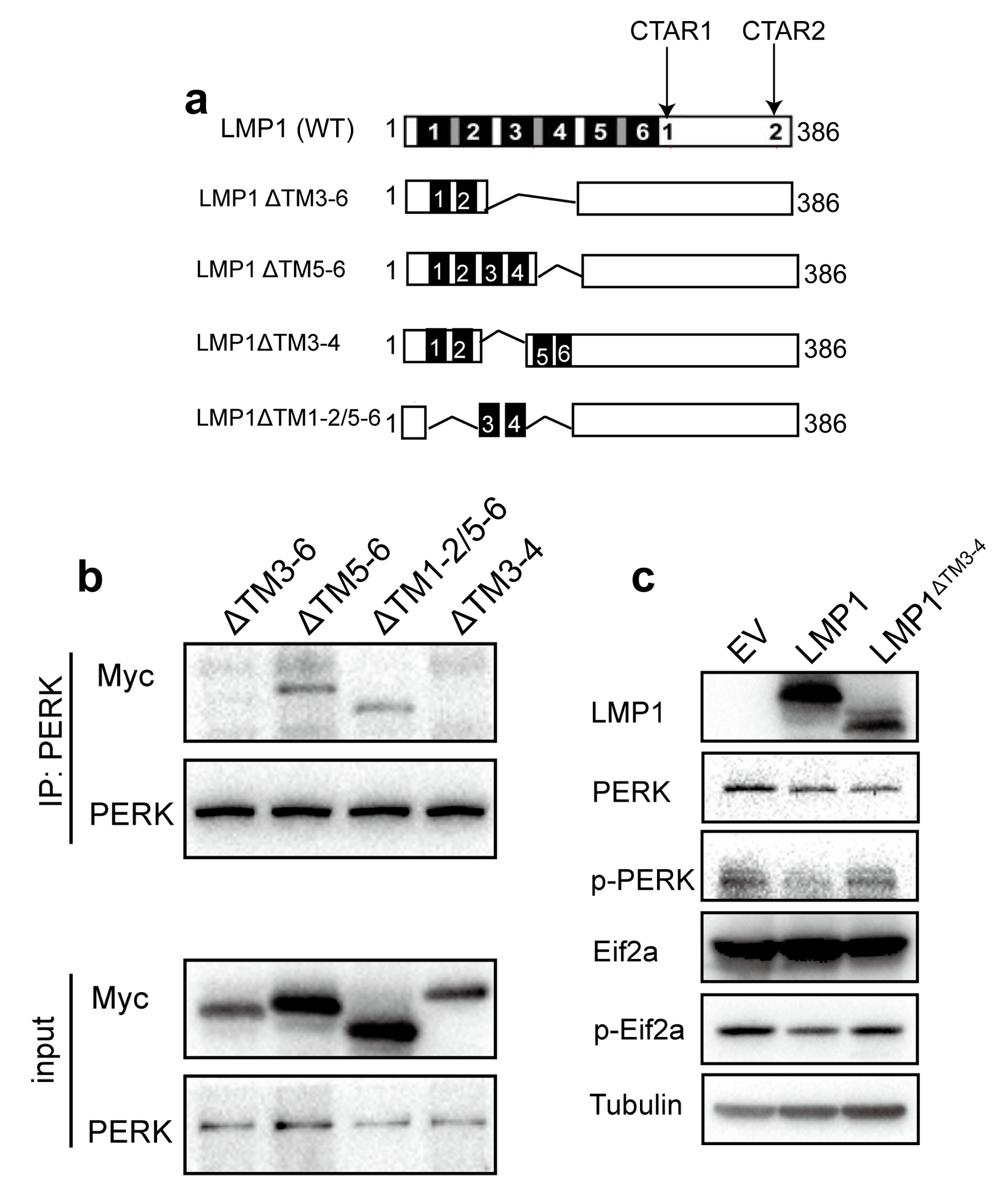
**

**Supplementary Fig. 3. The LMP1 TM3-4 domain interacts with PERK.** (**a**) A schematic diagram of the LMP1 deletion mutants used in this study is shown. (**b**) HEK293T cells were transfected with the indicated myc-tagged mutants, followed by IP with an anti-PERK antibody to identify the domain responsible for the PERK interaction. (**c**) CNE1 cells were stably transfected with the indicated plasmid, followed by immunoblotting to detect PERK activity.

**
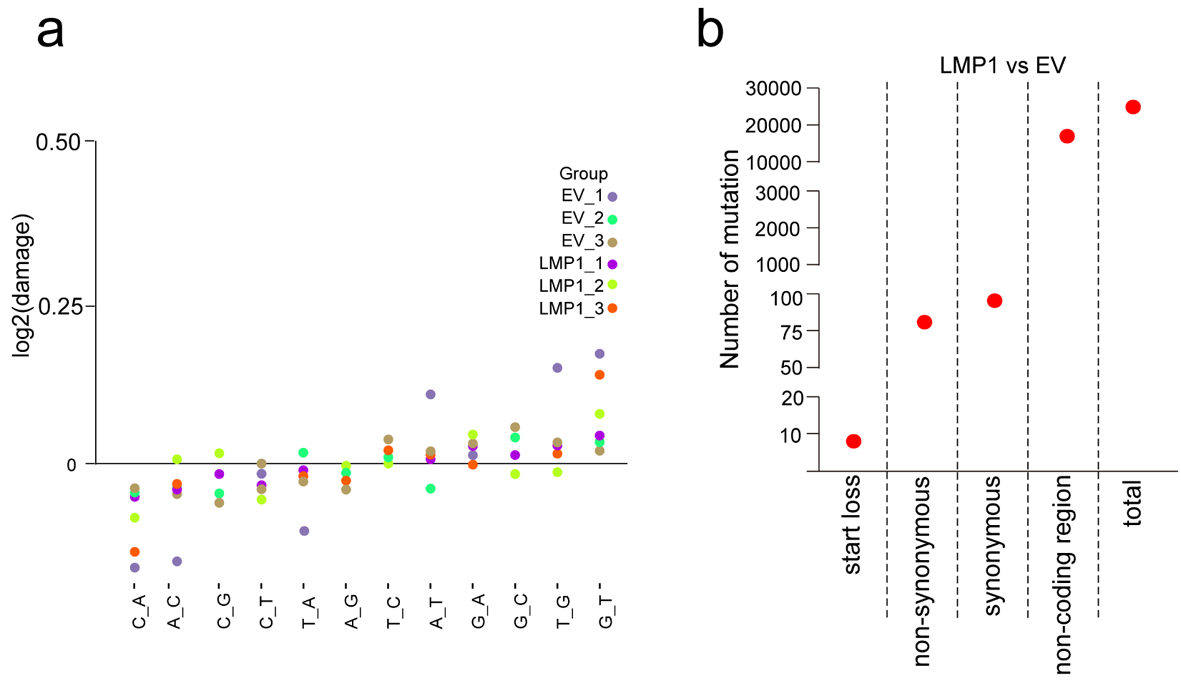
**

**Supplementary Fig. 4. LMP1 induced DNA mutation.** (**a**,**b**) Type of mutation (**a**) and number of mutations (**b**) of LMP1-overexpression cells and controls determine by whole-exome sequencing.

**
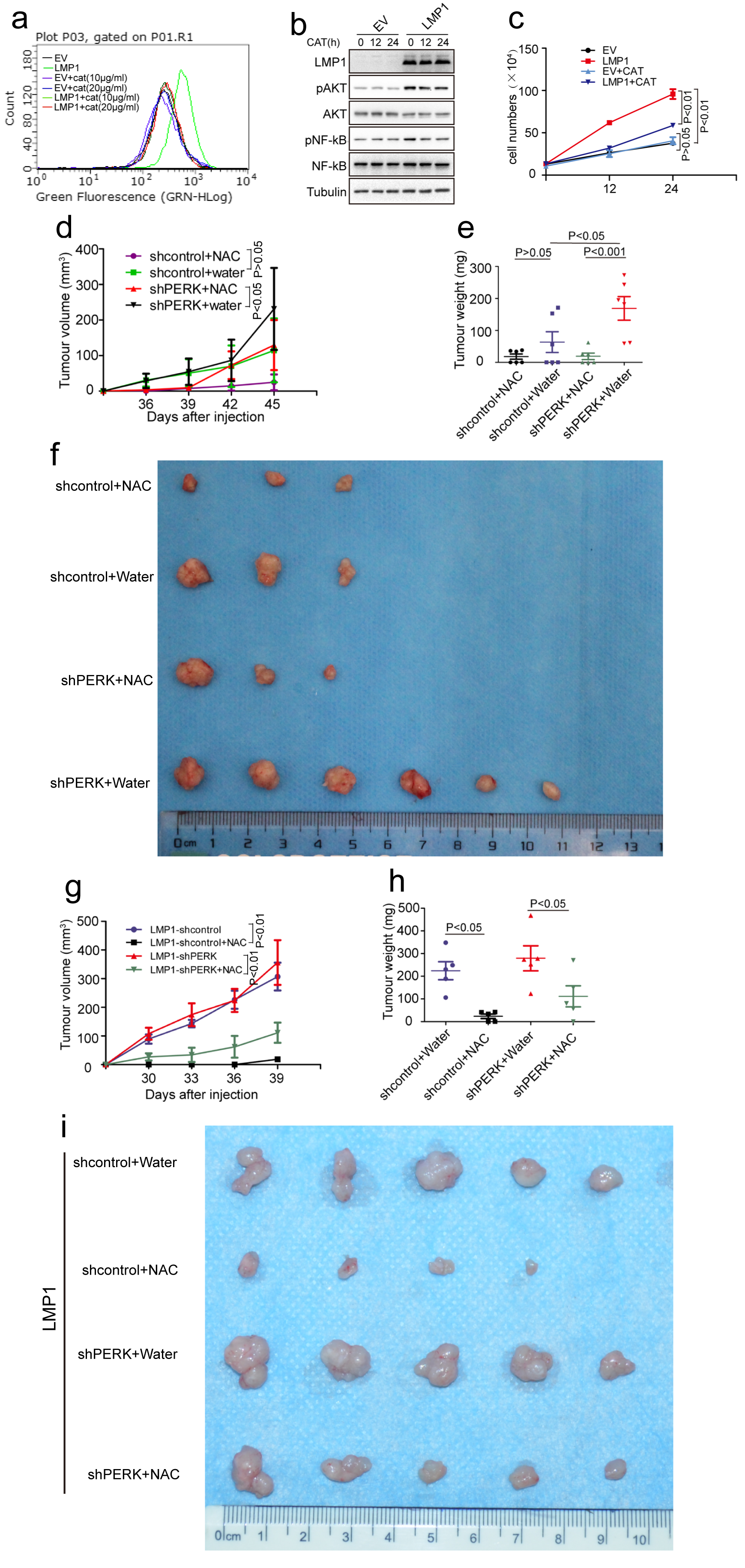
**

**Supplementary Fig. 5. LMP1 promotes tumor growth by modulating PERK-mediated ROS production.** (**a**) CNE1-EV or CNE1-LMP1 cells were treated with catalase at 24 h, followed by staining with CM-H2DCFDA, and oxidative stress was analyzed by flow cytometry. (**b**) Immunoblotting was performed to analyze p-AKT, AKT, p-NF-KB, NF-KB, and tubulin in CNE1-EV or CNE1-LMP1 cells treated with catalase for the indicated times. (**c**) CNE1-EV or CNE1-LMP1 cells were treated with catalase for 24 h, followed by examination of cell proliferation by a CCK8 assay. (**d**) CNE1 cells transduced with the indicated shRNA (2.5×10^6^) were subcutaneously transplanted into nude mice, which received water with or without 40 mM N-acetylcysteine (NAC). Tumors were collected on day 45, and tumor volume (**d**) and weight (**e**) were quantified. (**g**) CNE1-LMP1 cells transduced with the indicated shRNA (2.5×10^6^) were subcutaneously transplanted into nude mice, which received water with or without 40 mM NAC. Tumors were collected on day 39, and tumor volume (**g**) and weight (**h**) were quantified. n=5 mice per group, the data are presented as the mean ± SE. * P< 0.05.


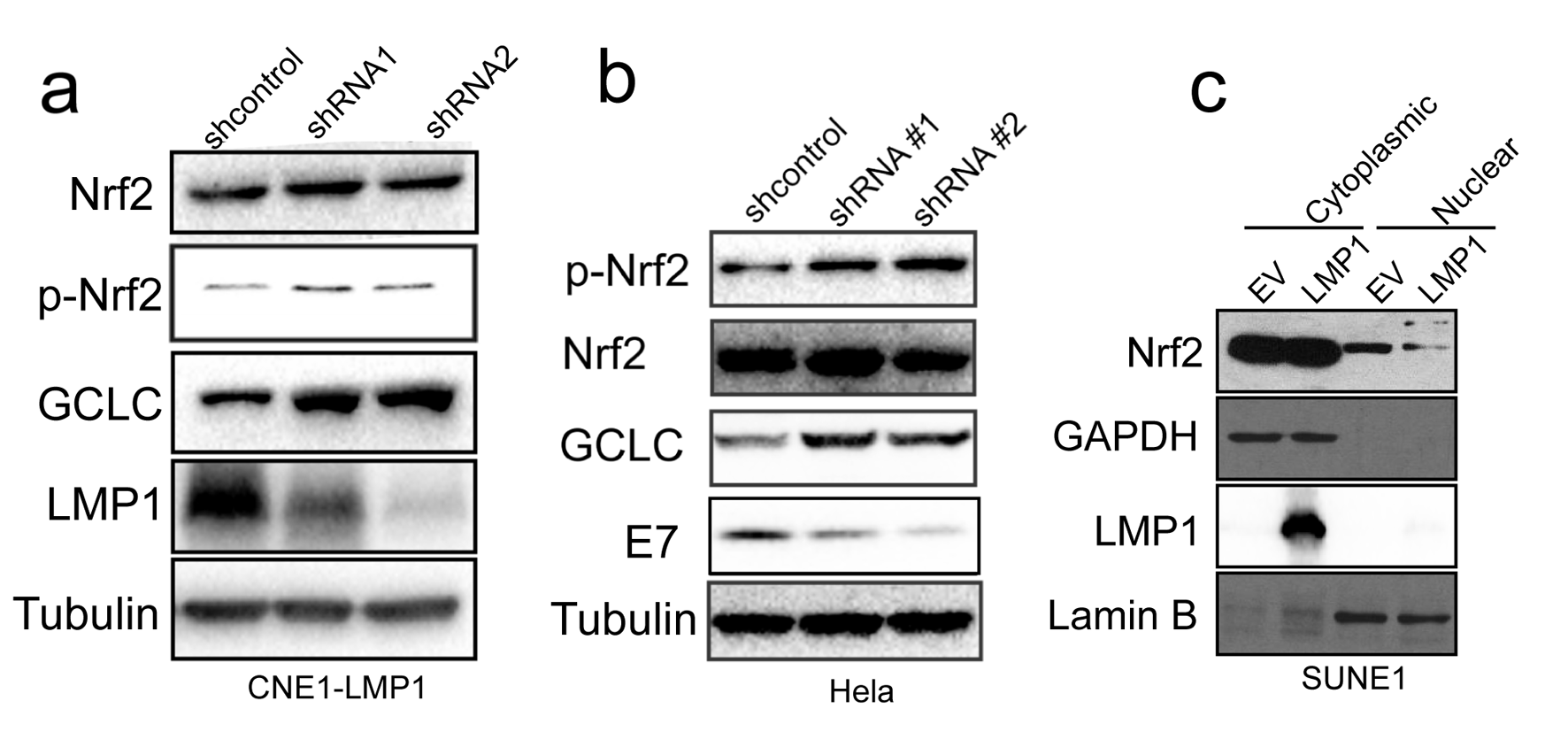


**Supplementary Fig. 6. DNA tumor virus oncogenes inhibit Nrf2-mediated antioxidant response**. (**a**) Immunoblotting of GCLC, Nrf2, p-Nrf2, LMP1 and Tubulin in CNE1-LMP1 cells transduced with two independent LMP1 shRNAs. (**b**) Immunoblotting of GCLC, Nrf2, p-Nrf2 and Tubulin in Hela cells transduced with two independent E7 shRNAs. (c) Immunoblotting of Nrf2 in cytoplasmic and nuclear fractions of LMP1-transduced SUNE1 cells. GAPDH and Lamin B were used as cytoplasmic and nuclear markers.

**
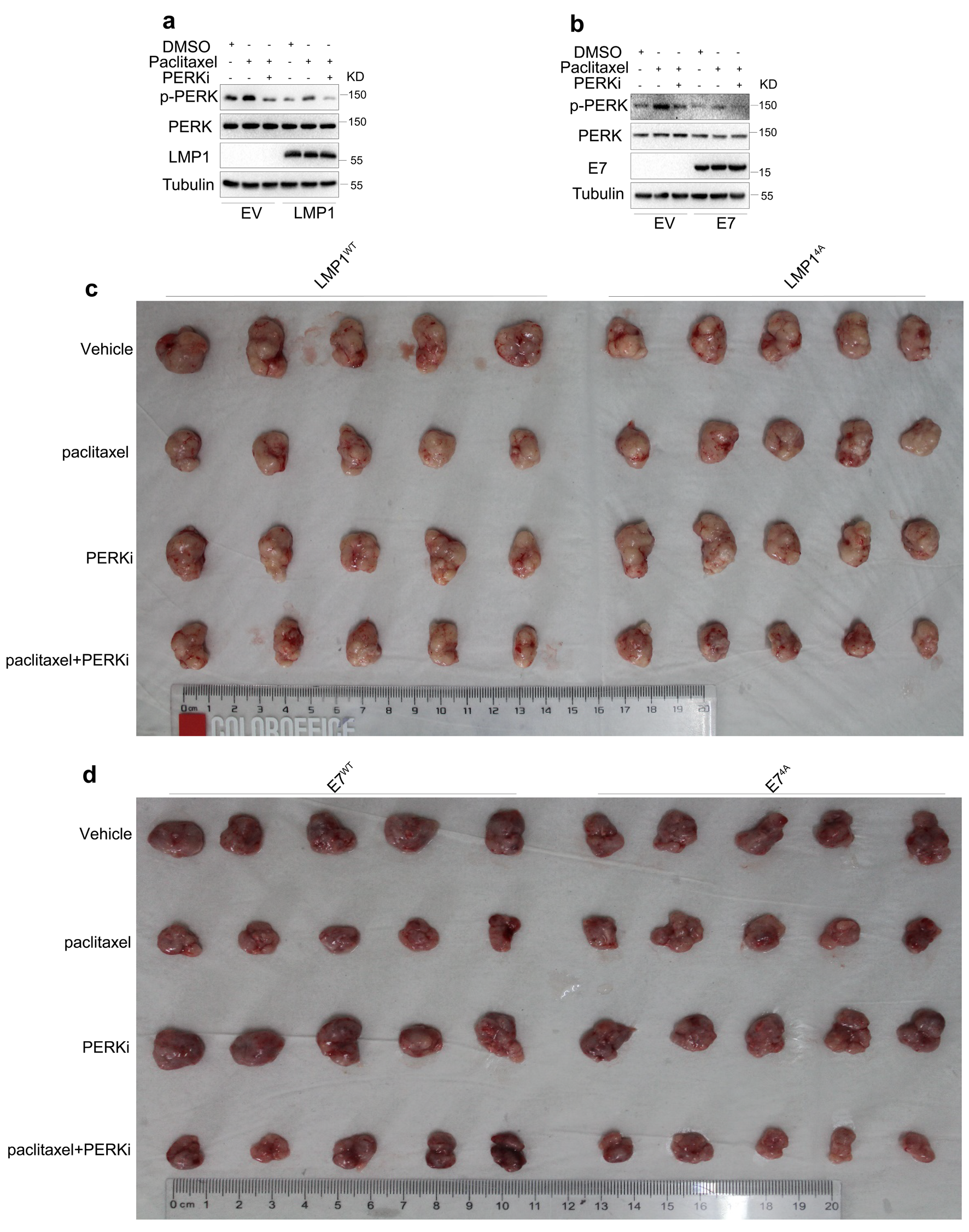
**

**Supplementary Fig. 7. DNA tumor virus oncogenes increase chemosensitivity**. (**a**,**b**) An aliquot of cells used in Figure 7a-c was analyzed by immunoblotting to detect the expression of viral oncoproteins and the activity of PERK. (**c**,**d**) tumor images of mice with subcutaneous injection of CNE1cells with LMP1^WT^ or LMP1^4A^ (**c**) and C33A cells with E7^WT^ or E7^4A^ (**d**).

**Table S1. siRNA sequence used in this study**

| Gene name | Target sequence(5 'to 3') |
| --- | --- |
| Control | TTCTCCGAACGTGTCACGT |
| E7 | GAAAACGATGAAAUAGATG |
| E6 | GCATGCTGCATGCCATAAA |
| E5 | CTGCCACAGCATTTACAGT |
| L1 | GCTGGCAGCTCTAGATTAT |
| L2 | GCACCTAGGCCTACGTTTA |
| PERK | GATTAAGGGTGCAACTAAA |
|  | CGAAGAATCTTCCGAAGAA |
